# Enrollment Characteristics and Risk Behaviors of Injection Drug Users Participating in the Bangkok Tenofovir Study, Thailand

**DOI:** 10.1371/journal.pone.0025127

**Published:** 2011-09-28

**Authors:** Michael Martin, Suphak Vanichseni, Pravan Suntharasamai, Udomsak Sangkum, Rutt Chuachoowong, Philip A. Mock, Manoj Leethochawalit, Sithisat Chiamwongpaet, Somyot Kittimunkong, Frits van Griensven, Janet M. McNicholl, Lynn Paxton, Kachit Choopanya, for the Bangkok Tenofovir Study Group

**Affiliations:** 1 Thailand Ministry of Public Health – US Centers for Disease Control and Prevention Collaboration, Nonthaburi, Thailand; 2 Centers for Disease Control and Prevention, Atlanta, Georgia, United States of America; 3 Bangkok Tenofovir Study Group, Bangkok, Thailand; 4 Bangkok Metropolitan Administration, Bangkok, Thailand; 5 Thailand Ministry of Public Health, Nonthaburi, Thailand; Vanderbilt University, United States of America

## Abstract

**Background:**

The Bangkok Tenofovir Study was launched in 2005 to determine if pre-exposure prophylaxis with tenofovir will reduce the risk of HIV infection among injecting drug users (IDUs). We describe recruitment, screening, enrollment, and baseline characteristics of study participants and contrast risk behavior of Tenofovir Study participants with participants in the 1999–2003 AIDSVAX B/E Vaccine Trial.

**Methods:**

The Bangkok Tenofovir Study is an ongoing, phase-3, randomized, double-blind, placebo-controlled, HIV pre-exposure prophylaxis trial of daily oral tenofovir. The Tenofovir Study and the Vaccine Trial were conducted among IDUs at 17 drug-treatment clinics in Bangkok. Tenofovir Study sample size was based on HIV incidence in the Vaccine Trial. Standardized questionnaires were used to collect demographic, risk behavior, and incarceration data. The Tenofovir Study is registered with ClinicalTrials.gov, number-NCT00119106.

**Results:**

From June 2005 through July 2010, 4094 IDUs were screened and 2413 enrolled in the Bangkok Tenofovir Study. The median age of enrolled participants was 31 years (range, 20–59), 80% were male, and 63% reported they injected drugs during the 3 months before enrollment. Among those who injected, 53% injected methamphetamine, 37% midazolam, and 35% heroin. Tenofovir Study participants were less likely to inject drugs, inject daily, or share needles (all, p<0.001) than Vaccine Trial participants.

**Discussion:**

The Bangkok Tenofovir Study has been successfully launched and is fully enrolled. Study participants are significantly less likely to report injecting drugs and sharing needles than participants in the 1999–2003 AIDSVAX B/E Vaccine Trial suggesting HIV incidence will be lower than expected. In response, the Bangkok Tenofovir Study enrollment was increased from 1600 to 2400 and the study design was changed from a defined 1-year follow-up period to an endpoint-driven design. Trial results demonstrating whether or not daily oral tenofovir reduces the risk of HIV infection among IDUs are expected in 2012.

## Introduction

HIV spread rapidly among injecting drug users (IDUs) in Bangkok in the late 1980s [1] and HIV prevalence has remained high, 30% to 50% [2], [3]. Safe and effective tools to prevent HIV infection among IDUs are urgently needed. Use of antiretroviral drugs before HIV exposure (pre-exposure prophylaxis) may protect people at high risk of HIV from infection and provide a new tool to reduce HIV transmission.

The Bangkok Tenofovir Study is an ongoing phase-3, randomized, double-blind, placebo-controlled, endpoint-driven HIV prevention trial that aims to determine if daily oral Tenofovir Disoproxil Fumarate (tenofovir) will reduce HIV transmission among IDUs. Tenofovir, a nucleotide reverse transcriptase inhibitor, is a potent antiretroviral with a long half-life allowing once-daily dosing [4]–[7]. Data from clinical trials among people infected with HIV have shown that tenofovir has a good safety profile [8], [9], a low potential to select for tenofovir resistance [10], [11], and co-administration of tenofovir with methadone and/or oral contraceptives does not alter the pharmacokinetics or pharmacodynamics of these drugs [12], [13]. Tenofovir was licensed for the treatment of HIV infection by the US Food and Drug Administration in 2001 and the Thailand Food and Drug Administration in 2006.

Several lines of evidence suggest that pre-exposure prophylaxis with tenofovir will reduce HIV transmission among IDUs. Studies of macaques have shown that tenofovir can prevent or delay infection with simian immunodeficiency virus and humanized derivatives called SHIV [14]–[17] and the use of antiretroviral drugs reduces the risk that HIV-infected pregnant women will transmit HIV to their newborns [18] and that health care workers will become infected following occupational exposures to HIV [19], [20].

Following consultations with IDUs and representatives of organizations working with communities at risk for HIV infection, we began preparations for an HIV pre-exposure prophylaxis trial in 2004 [21]. The findings of two longitudinal studies conducted among IDUs in Bangkok informed the design of the study. The first was a preparatory cohort study [22] that enrolled 1209 IDUs at drug-treatment clinics managed by the Bangkok Metropolitan Administration (BMA), the city government of Bangkok, during 1995–1996 and followed them for 3 years. The second study was the 1999–2003 AIDSVAX B/E Vaccine Trial [23] conducted among 2546 IDUs in the same BMA drug-treatment clinics. The vaccine did not prevent HIV infection, but IDUs showed a continued willingness to participate in research and 90% completed the study. HIV incidence remained stable during follow-up at 3.4 per 100 person-years.

Based on community interest in promising HIV prevention interventions, ongoing high HIV incidence among IDUs [23], and evidence suggesting pre-exposure prophylaxis with tenofovir would prevent HIV infection [14]–[20], a protocol to evaluate tenofovir among IDUs was developed and submitted for regulatory review. Regulatory approvals were obtained in May 2005 and the Bangkok Tenofovir Study was launched in June 2005.

Since the study began several other pre-exposure prophylaxis trials have provided results that support the rationale for the Bangkok Tenofovir Study. A trial among women in South Africa found use of tenofovir vaginal gel reduced HIV acquisition by 39% [24] and a trial among men who have sex with men found use of daily oral truvada (tenofovir+emtricitabine) reduced HIV incidence by 44% [25]. Although a trial comparing daily oral truvada to placebo among women in several African countries was stopped in 2010 because interim data showed that it was unlikely the study would demonstrate lower HIV infection rates among women receiving truvada [26], a trial among heterosexual men and women in Botswana found that participants randomized to receive daily truvada were 63% less likely to become HIV infected than participants receiving placebo [27] and a trial among heterosexual HIV serodiscordant couples in Kenya and Uganda found that daily tenofovir reduced HIV acquisition 62% and daily truvada 73% compared to placebo [28].

Overall, these studies provide evidence that pre-exposure prophylaxis with tenofovir or truvada is safe and can reduce the risk of sexual transmission of HIV among heterosexual couples and men who have sex with men. The results do not provide information about the efficacy of tenofovir or truvada to prevent parenteral transmission of HIV among IDUs. In order to determine if daily oral tenofovir can reduce the risk of HIV infection among IDUs, we are moving forward to complete the Bangkok Tenofovir Study.

The research team became aware during the first year of the study that participant risk behavior differed from Vaccine Trial participants, suggesting HIV incidence would be lower than expected. Here, we describe Bangkok Tenofovir Study recruitment, screening, and enrollment, and contrast risk behavior of Tenofovir Study participants with Vaccine Trial participants. These findings led the research team to increase Bangkok Tenofovir Study enrollment and change to an endpoint-driven study design.

## Methods

The protocol for this trial and supporting CONSORT checklist are available as supporting information; see Checklist S1 and Protocol S1.

### Community engagement

In preparation for the study, the research team met with IDUs and representatives of organizations working with communities at risk for HIV infection, to describe the project; distribute draft protocols, consent forms, and education materials; and to gather input for trial materials and procedures. Focus group discussions were conducted to assess IDU willingness to join an HIV pre-exposure prophylaxis study, understanding of clinical trial design and procedures, and concerns about the use of tenofovir. A community relations committee made up of at least one IDU from each of the 17 BMA clinics was formed to provide ongoing community input during the trial. The committee meets with the research team every 2 months to discuss a broad range of issues that impact IDUs, clinic staff, and the study.

### Study setting and design

The study is being conducted at 17 BMA drug-treatment clinics in the densely populated urban communities of Bangkok. The clinics offer a range of services including HIV counseling and testing, risk-reduction counseling, social and welfare services, health education, primary medical care and referrals, methadone treatment, condoms, and bleach to clean injection equipment with demonstrations of appropriate use. These services are free of charge. Thailand's narcotics law prohibits the distribution of needles to inject illicit drugs and needles are not provided in the clinics; however, sterile needles and syringes are available to the public over the counter at low cost (5 to 10 baht/0.12 to 0.25 USD) in pharmacies in Bangkok.

We based sample size calculations for the Tenofovir Study on HIV incidence among IDUs in the 1999–2003 Vaccine Trial [23]. We estimated the efficacy of tenofovir to prevent HIV infection was 67% and designed the study to have 80% power to demonstrate at least 10% tenofovir efficacy with a one-sided type 1 error of 2.5%. In order to meet these criteria, we planned to enroll 1600 participants and follow each participant for 12 months. During the first year of the Tenofovir Study we discovered that participant reports of injecting and needle sharing were less than observed in the Vaccine Trial, suggesting HIV incidence would be lower than expected. Based on the original Tenofovir Study design, a lower than expected HIV incidence would have resulted in fewer endpoints (i.e., incident HIV infections), a larger confidence interval around the efficacy estimate, and a diminished likelihood the trial would determine if daily oral tenofovir reduced the risk of HIV infection among IDUs. In response, the Bangkok Tenofovir Study enrollment target was increased from 1600 to 2400 and the study design was changed from a defined 1-year follow-up period to an endpoint (incident HIV infection)-driven design. These changes provide 82% power to demonstrate at least 10% tenofovir efficacy with a one-sided type 1 error of 2.5%.

### Regulatory review

The study protocol, informed consent documents, questionnaires, and education materials were reviewed and approved by the BMA Ethical Review Committee, the Thailand Ministry of Public Health Ethical Review Committee, and an Institutional Review Board of the US Centers for Disease Control and Prevention. The US Office for Human Research Protections approved the follow-up of incarcerated participants.

An independent Data and Safety Monitoring Board conducts annual safety reviews and one interim efficacy review. A clinical research organization is employed to provide independent oversight and assure compliance with international guidelines for good clinical practice. Gilead (Gilead Sciences, Inc., Foster City, California) provides tenofovir and placebo free of charge but has not been involved in the design or conduct of the study, data analysis, or the presentation of results.

### Recruitment

Research staff placed posters and brochures describing the study in the drug-treatment clinics, provided presentations about the study at IDU drop-in centers, and were available at the clinics to discuss the study with individuals interested in joining the trial. Potential participants received an explanation of study objectives and design, eligibility criteria, and study activities and procedures.

### Eligibility evaluation

HIV-uninfected individuals aged 20 to 60 years who reported injecting drugs during the previous 12 months were candidates for the study. Table 1 lists eligibility criteria. We excluded people with chronic HBV infection because of concerns about reactivation (i.e., flares) of hepatic disease if use of tenofovir was stopped. HBV vaccine is provided to enrolled participants who have no serologic evidence of active or chronic Hepatitis B infection. We excluded women who were pregnant or breast feeding and required women to agree to abstain from sexual intercourse or use contraception (i.e., oral, injection, or barrier) during the study because large well-controlled studies of tenofovir had not been conducted among pregnant or breast-feeding women. Contraceptives are provided to participants free of charge.

**Table 1:** Eligibility requirements for the Bangkok Tenofovir Study.

In order to enroll potential participants must:	
•	Be 20 to 60 years-old
•	Report injection drug use in the 12 months before screening
•	Possess documentation of a Thai National Identification number
Have the following laboratory results from a blood or oral fluid specimen collected in the 2 weeks before enrollment:	
•	A non-reactive HIV oral fluid test
•	Hemoglobin ≥9 gm/dL
•	Alanine aminotransferase (ALT) ≤102 U/L
•	Aspartate aminotransferase (AST) ≤95 U/L
•	Total bilirubin ≤1.5 mg/dL
•	Amylase ≤144 U/L
•	Phosphorus ≥2.2 mg/dL
•	Negative hepatitis B surface antigen
•	Calculated creatinine clearance ≥60 mL/min by the Cockcroft-Gault formula where creatinine clearance in mL/min = Male: (140−age in years)×(wt in kg)/72×(serum creatinine in mg/dL)Female: (140−age in years)×(wt in kg)×0.85/72×(serum creatinine in mg/dL)
•	Pass the Bangkok Tenofovir Study comprehension test
•	Be willing and able to provide informed consent for study participation
•	Be available and committed to daily or monthly follow-up for at least 12 months
In addition:	
•	Women must not be pregnant or breastfeeding and must be willing to abstain from sexual intercourse or use contraception during the trial (i.e., oral, injection, or barrier)
•	A volunteer may be excluded if s/he has a history of significant renal, liver, or bone disease or other clinical condition or prior therapy that, in the judgment of the study physician, would make the subject unsuitable for the study
•	A volunteer will be excluded if s/he is participating in another HIV prevention, drug, or vaccine trial

Eligible volunteers completed a comprehension test to assess understanding of key trial concepts. Volunteers meeting all eligibility criteria were enrolled after providing written informed consent.

### Enrollment and randomization

Enrolled participants were randomly assigned in a 1∶1 ratio to receive daily oral tenofovir 300 mg or placebo. The randomization list, created using a computerized random-number generator, was shared with Gilead who prepared and labeled bottles with a randomization number. When an eligible participant completed the consent process, study staff assigned them the next sequential randomization number. Participants, study staff, monitors, and other staff involved in the trial are blinded to drug assignment for the duration of the study.

Research staff collected baseline demographic and incarceration information using interviewer-administered questionnaires. Participants chose daily clinic visits with directly observed taking of study drug (DOT) or monthly visits without DOT. At enrollment and each monthly follow-up visit, participants are assessed for adverse events, oral fluid is collected for HIV testing (OraQuick Rapid HIV-1/2 Antibody Test, assembled in Thailand for Orasure Technologies, Inc, Bethlehem, Pennsylvania) and urine for pregnancy testing (OneStep urine test, ULTI Med Products, Ahrensburg, Germany); adherence is assessed using an audio computer-assisted self-interview (ACASI) and pill counts; and adherence and HIV risk-reduction counseling are provided. Reactive oral fluid HIV tests are confirmed using two different enzyme-immunoassays on blood (EIA) (Genetic Systems HIV-1/HIV-2 EIA, Washington, USA) and Western blot (Bio-Rad, Redmond, Washington).

At enrollment, months 1, 2, 3, and every 3 months thereafter, blood is collected for hematologic, hepatic, and renal safety assessment. Participants complete a risk questionnaire assessing drug use, incarceration, and sexual activity during the previous 3 months using an ACASI at enrollment and every 3 months thereafter.

To compensate participants for their time, effort, and travel, they receive 350 baht (∼10 US dollars) for each monthly study visit. Participants on the DOT schedule receive 70 baht (∼2 US dollars) each day they come to clinic and participants who come all 7 days in a week receive 350 baht for that week.

### AIDSVAX B/E Vaccine Trial

The AIDSVAX B/E Vaccine Trial was a phase-3, randomized, double-blind, placebo-controlled study conducted among IDUs at BMA drug-treatment clinics during 1999–2003. HIV-uninfected individuals aged 20 to 60 years who reported injecting drugs during the previous 12 months were eligible for the trial. Pregnant or breastfeeding women were excluded. There were no blood chemistry or hematologic eligibility requirements. Detailed descriptions of the study have been published [23], [29]–[33]. Briefly, 2546 IDUs enrolled and were randomly assigned (1∶1) to receive either AIDSVAX B/E or placebo at months 0, 1, 6, 12, 18, 24, and 30. An interviewer-administered questionnaire was used at baseline and every 6 months to assess drug use, incarceration, and sexual activity during the previous 6 months.

### Statistical analysis

Vaccine Trial and Tenofovir Study participant enrollment demographic and risk characteristics were compared using chi-square for categorical variables and *t-*test or Wilcoxon rank sum test for continuous variables. Adjusted odds ratios and 95% confidence intervals (CI) were estimated using logistic regression; factors significant (p<0.1) in univariate analyses were retained in the multivariable model. In order to evaluate injection of heroin, midazolam, and methamphetamine, we included these variables in the multivariable model and did not include ‘injected any drugs’.

We used SAS version 9.2 (SAS Institute, Cary, North Carolina) for statistical analyses.

## Results

### Baseline characteristics

**Figure 1:**
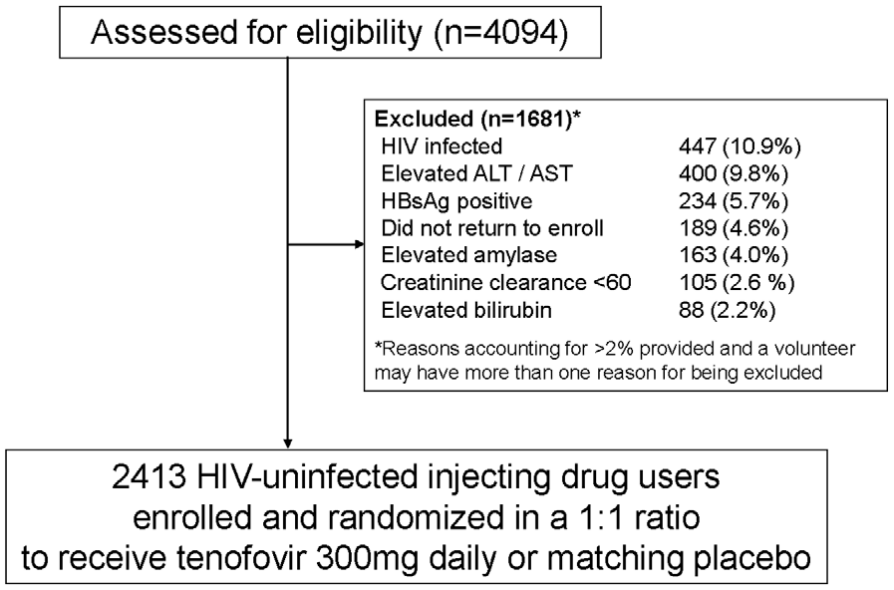
Number of injection drug users screened and enrolled in the Bangkok Tenofovir Study in Bangkok, Thailand, 2005–2010.

From June 2005 through July 2010, 4094 people were evaluated for enrollment (Figure 1). Their median age was 32 years (mean 33.3; range, 19–60), 82% were male and 11% were HIV infected. HIV infection was the most common reason screened volunteers were not able to enroll, followed by elevated AST or ALT (10%), and current or chronic hepatitis B infection (6%).

Among the 2413 (59%) IDUs who enrolled, the median age was 31 years (mean 32.4; range, 20–59), 80% were male, 48% had a primary school (grade 6) or less education, and 22% were in a methadone treatment program (Table 2). Most participants reported they had been incarcerated in the past: 1905 (79%) in a police holding cell, 1403 (58%) in prison, and 1386 (57%) in both. Participants reporting incarceration in police holding cells had been incarcerated a median of 3 times (range, 1–60); in prisons a median of 2 times (range, 1–20).

**Table 2:** Results of bivariate and multivariate analysis comparing baseline demographic characteristics and risk activities reported by injection drug users participating in the 1999–2003 AIDSVAX B/E Vaccine Trial and the ongoing Bangkok Tenofovir Study, Thailand.

		Vaccine	Tenofovir				
		N = 2546	N = 2413	OR		Adjusted OR	
Variable		No. (%)	No. (%)	(95% CI)	P value	(95% CI)	P value
Sex	Male	2377 (93.4)	1924 (79.7)	0.3 (0.2–0.3)	<0.001	0.4 (0.3–0.5)	<0.001
	Female	169 (6.6)	489 (20.3)	1.0		1.0	
Age (years)	Mean	28.8	32.4	1.0 (1.0–1.1)	<0.001	1.1 (1.1–1.1)	<0.001
Education	≤Primary (year 6)	833 (32.7)	1154 (47.8)	1.9 (1.7–2.1)	<0.001	1.1 (0.9–1.3)	0.39
	Secondary or more	1713 (67.3)	1259 (52.2)	1.0		1.0	
Ever been in jail (holding cell)	Yes	1943 (76.3)	1905 (79.0)	1.2 (1.0–1.3)	0.03	1.3 (1.0–1.8)	0.05
	No	603 (23.7)	508 (21.0)	1.0		1.0	
Ever been in prison	Yes	1278 (50.2)	1403 (58.1)	1.4 (1.2–1.5)	<0.001	1.0 (0.8–1.2)	0.86
	No	1268 (49.8)	1010 (41.9)	1.0		1.0	

Reported risks*		N = 2546	N = 2405				
In methadone treatment	Yes	2150 (84.4)	523 (21.8)	0.05 (0.04–0.06)	<0.001	0.1 (0.1–0.2)	<0.001
	No	396 (15.6)	1882 (78.2)	1.0			
Injected any drugs	Yes	2389 (93.8)	1506 (62.6)	0.1 (0.1-0.1)	<0.001	Not included	
	No	157 (6.2)	899 (37.4)	1.0			
Injected heroin	Yes	2355 (92.5)	526 (21.9)	0.02 (0.02–0.03)	<0.001	0.05 (0.04–0.06)	<0.001
	No	191 (7.5)	1879 (78.1)	1.0		1.0	
Injected methamphetamine	Yes	388 (15.2)	801 (33.3)	2.8 (2.4–3.2)	<0.001	2.7 (2.1–3.3)	<0.001
	No	2158 (84.8)	1604 (66.7)	1.0		1.0	
Injected midazolam	Yes	236 (9.3)	558 (23.2)	3.0 (2.5–3.5)	<0.001	7.7 (5.9–10.0)	<0.001
	No	2310 (90.7)	1847 (76.8)	1.0		1.0	
Injection frequency	Daily	936 (36.8)	204 (8.5)	0.2 (0.1–0.2)	<0.001	0.5 (0.4–0.7)	<0.001
	<Daily	1610 (63.2)	2201 (91.5)	1.0		1.0	
Shared needles	Yes	790 (31.0)	435 (18.1)	0.5 (0.4–0.6)	<0.001	0.6 (0.5–0.8)	<0.001
	No	1756 (69.0)	1970 (81.9)	1.0		1.0	
In jail (police holding cell)	Yes	360 (14.1)	552 (23.0)	1.8 (1.6–2.1)	<0.001	1.4 (1.0–1.8)	0.03
	No	2186 (85.9)	1853 (77.0)	1.0			
In prison	Yes	188 (7.4)	389 (16.2)	2.4 (2.0–2.9)	<0.001	1.2 (0.8–1.6)	0.45
	No	2358 (92.6)	2016 (83.8)	1.0			
Sexual intercourse with more than one partner	Yes	306 (12.0)	529 (22.0)	2.1 (1.8–2.4)	<0.001	2.0 (1.5–2.5)	<0.001
	No	2240 (88.0)	1876 (78.0)	1.0		1.0	

OR = odds ratio; CI = confidence interval.

*Vaccine Trial participants reported risks for the 6 months before enrollment while Tenofovir Study participants reported risks for the 3 months before enrollment.

Baseline risk behavior data were available on 2405 (99.7%) of enrolled participants. At enrollment, 1506 (63%) participants reported they had injected drugs and 435 (18%) had shared needles during the 3 months before enrollment. Among those who injected, 801 (53%) injected methamphetamine, 558 (37%) midazolam, 526 (35%) heroin, 124 (8%) other sedative-hypnotics, and 55 (4%) other drugs. A substantial proportion of those who injected drugs reported injecting more than one drug: 203 (14%) injected methamphetamine and midazolam, 186 (12%) methamphetamine and heroin, 184 (12%) heroin and midazolam, and 69 (5%) heroin, methamphetamine, and midazolam.

Among the 610 (25%) participants who reported incarceration during the 3 months before enrollment, 552 (90%) had been in a police holding cell, 389 (64%) in prison, and 331 (54%) in both. Of those who spent time in holding cells, 40 (7%) reported injecting drugs and 36 (6%) reported sexual intercourse in the cells. Of those who had been in prison, 33 (8%) reported injecting drugs and 35 (9%) reported sexual intercourse in prison.

At enrollment, 682 (28%) participants reported they had not had sexual intercourse with a same sex or opposite sex partner during the previous 3 months; 1194 (50%) reported intercourse with one partner; and 529 (22%) with more than one partner. Among enrolled participants, 1044 (43%) reported sexual intercourse with a partner with whom they lived, 78 (8%) using a condom every time; 913 (38%) reported intercourse with at least one casual (i.e., non-live-in) partner, 299 (37%) of the 806 male participants used a condom every time. A data entry problem limited assessment of condom use with casual partners to men. Among the 1916 male participants who completed a risk assessment at enrollment, 91 (5%) reported sexual intercourse with at least one male partner and 44 (48%) of these men used a condom every time. Most, 73 (80%), of these men also reported sexual intercourse with women.

Bivariate and multivariable analyses comparing baseline characteristics of Vaccine Trial and Tenofovir Study participants are shown in Table 2. Tenofovir Study participants are considerably less likely to inject drugs than Vaccine Trial participants (odds ratio 0.1, 95% CI 0.1-0.1, p<0.001). Multivariable analyses shows that Tenofovir Study participants are more likely to be female and older than Vaccine Trial participants (both, p≤0.001). Tenofovir Study participants are less likely to have injected heroin but more likely to have injected midazolam or methamphetamine than Vaccine Trial participants (all, p<0.001). Tenofovir Study participants are less likely to inject daily (p<0.001) or share needles (p = <0.001) but more likely to report sexual intercourse with more than one partner (p<0.001) than Vaccine Trial participants.

## Discussion

The Bangkok Tenofovir Study, an ongoing HIV pre-exposure prophylaxis trial among IDUs, has been successfully launched and is fully enrolled. Participant reports of injection drug use and needle sharing are significantly less than expected, suggesting HIV incidence will be lower than incidence estimates used in the design of the study. To improve the likelihood the trial achieves its primary objective, to determine if daily oral tenofovir prevents HIV infection, we increased the enrollment target from 1600 to 2400 and changed participant follow-up from a defined 1-year follow-up period to an endpoint-driven design.

The lower level of risk behavior reported by Tenofovir Study participants compared to Vaccine Trial participants is likely due to several reasons. Risk-reduction counseling, methadone treatment, and other services offered by the BMA drug-treatment clinics may have reduced risk behavior among IDUs attending the clinics. Tenofovir Study eligibility requirements including blood chemistry and hematologic assessments and the exclusion of individuals with chronic hepatitis B infection may have led to the enrollment of a population of participants who inject drugs and share needles less frequently than participants in the Vaccine Trial. Changes in drugs injected by Vaccine Trial participants were recognized during the 1999–2003 trial [32], [33]. A decrease in heroin use, which is usually injected, and more methamphetamine and midazolam use, which can be taken orally or inhaled, may be contributing to lower levels of injection drug use and needle sharing. These changes coincided with the Thai Government's ‘War on Drugs’. The drug war was launched in 2003 to decrease the supply of illicit drugs [34], [35]. During 2003, the supply of heroin in Bangkok decreased and the price increased four-fold from approximately 2500 to 10,000 Thai baht (60 to 250 USD) per 1000 mg [32]. The price of methamphetamine and midazolam increased as well, but remained more affordable: methamphetamine 150–250 baht (4 to 6 USD) per tablet and midazolam 40–60 baht (1 to 1.5 USD) per tablet. The changes in drug use are likely due in part to the changes in drug supply and cost.

It has taken 5 years to enroll 2413 IDUs in the Tenofovir Study compared to 18 months to enroll 2546 IDUs in the Vaccine Trial. More stringent Tenofovir Study inclusion criteria likely contributed to slower enrollment. There is also evidence that the number of IDUs in Bangkok has decreased in recent years. Researchers using capture-recapture methodology estimated there were 36,600 active opiate users in Bangkok in 1991 [36]. A population assessment done in 2003 using respondent-driven sampling methodology estimated there were 3595 IDUs in Bangkok [37]. Each methodology has limitations but the 10-fold difference suggests the population of IDUs in Bangkok has decreased.

Incarceration is a common experience among IDUs in Bangkok [29] with almost 80% of study participants reporting a history of incarceration and 25% incarcerated during the 3 months before enrollment. Previous studies have demonstrated an association between incarceration and HIV infection [29], [33], [38] and information about this association has been included in participant risk-reduction counseling.

Tenofovir Study participants report modest levels of sexual activity, with 78% reporting no sexual intercourse or intercourse with only one partner during the 3 months before enrollment. Study participants are, however, more likely to report sexual intercourse than Vaccine Trial participants. This change in sexual behavior may be related to decreasing heroin use and increasing methamphetamine use, warranting additional research [39], [40]. Previous studies among IDUs in Bangkok found no association between sexual activity and HIV infection [22], [33]. It will be important to monitor participant sexual activity to see if this remains true.

There are a number of limitations to this analysis including the use of self-reports. Self-reporting of stigmatized or illegal behavior is problematic and under-reporting of these activities is possible [41]. At baseline, Vaccine Trial participants reported risk behaviors for the previous 6 months using a standardized interviewer-administered questionnaire, while Tenofovir Study participants reported risk behaviors for the previous 3 months using ACASI. Studies suggest that ACASI provides an acceptable and more accurate method of collecting health risk behavior data than face-to-face interviews [42]–[44]. Tenofovir Study participants may more accurately report risk behaviors because of the shorter recall period (3 months in the Tenofovir Study, 6 months in the Vaccine Trial) and the use of ACASI. On the other hand, Vaccine Trial participants had a longer time at risk and may have been more willing to report risk behavior that took place more than 3 months ago [42]. The use of different data collection tools and time frames limits the comparability of risk behavior data from the two trials.

The Bangkok Tenofovir Study is fully enrolled. The Data Safety and Monitoring Board has recommended trial continuation following annual safety reviews and an interim efficacy review in 2009. Trial results demonstrating whether or not daily oral tenofovir is safe to use among HIV-uninfected IDUs and if tenofovir reduces the risk of HIV infection are expected in 2012.

## Supporting Information

Checklist S1 CONSORT Checklist.
(DOC)

Protocol S1 Trial Protocol.
(DOC)
