# Supplementary material for: Enrollment Characteristics and Risk Behaviors of Injection Drug Users Participating in the Bangkok Tenofovir Study, Thailand
Source: PLoS One. 2011 Sep 28;6(9):e25127. doi: 10.1371/journal.pone.0025127 (PMC3182181; doi:10.1371/journal.pone.0025127)
Supplement: Protocol S1 — Trial Protocol. (DOC) [file pone.0025127.s002.doc]

**PROTOCOL S1**

**Title:** Study of the Safety and Efficacy of Daily Tenofovir to Prevent HIV Infection Among Injection Drug Users in Bangkok, Thailand (Bangkok Tenofovir Study)

**Principal Investigator:** Dr. Kachit Choopanya

Bangkok Tenofovir Study Group

**Study Sponsor:** Epidemiology Branch

Division of HIV/AIDS Prevention-Surveillance and Epidemiology

National Center for HIV, STD, and TB Prevention

U.S. Centers for Disease Control and Prevention (CDC)

1600 Clifton Road, Mailstop E-45

Atlanta, GA 30333, USA

**Pharmaceutical Support:** Gilead Sciences, Inc.

333 Lakeside Drive

Foster City, CA 94404, USA

**Table of contents**

1. Bangkok Tenofovir Study Group ……………………………………………………… 5

1.1. Roles and responsibilities…………………………………………………………7

2. Protocol Summary……………………………………………………………………… 8

3. Abbreviations and Acronyms ………………………………………………………....10

4. Signature page………………………………………………………………………….12

5. Introduction……………………………………………………………………………. 13

5.1. Project justification

5.2. Why tenofovir?

5.3. Why a phase II safety study?

5.4. Potential increase in HIV associated risk behavior

5.5. Tenofovir adherence

6. Study objectives………………………………………………………………………. 15

6.1. Primary objectives

6.2. Secondary objectives

7. Trial design………………………………………………………………………....... 15

7.1. Primary endpoints

7.2. Secondary endpoints

8. Study design…………………………………………………………………………... 16

8.1. Study population

8.1.1. Recruitment

8.2. Inclusion criteria

8.3. Exclusion criteria

8.4. Participant retention

8.5. Participant withdrawal

8.6. Community relations committee

9. Study products………………………………………………………………………… 19

9.1. Tenofovir disoproxil fumarate

9.1.1. Chemical and pharmacokinetic properties

9.1.2. Clinical trials

9.1.3. Safety profile

9.2. Placebo

9.3. Drug accountability

9.4. Adherence counseling

9.5. Adherence monitoring

9.6. Study drug interruption

9.7. Concomitant medications

10. Study visit summary…………………………………………………………………... 23

10.1. Screening visit

10.2. Enrollment visit

10.3. Follow-up visits

10.3.1. Daily directly observed therapy

10.3.2. Monthly visits (scheduled at 4 week intervals)

10.3.3. First two monthly visits and three monthly visits (weeks 4, 8, 12, 24, 36, etc.)

10.3.4. Exit visit (Study completion, HIV infection confirmation visit, early termination)

10.3.5. Follow-up visits for newly HIV infected participants

10.3.6. Unscheduled visits

10.3.7. HIV and additional testing in scheduled and exit visits

11. Ethics ………………………………………………………………………………....... 27

11.1. Risks

11.2. Benefits

11.3. Post-trial access to effective products

11.4. Counseling

11.4.1. Pre-test counseling

11.4.2. Post-test counseling

11.4.3. HIV risk reduction counseling

11.5. Informed consent

11.6. Participant confidentiality

11.7. Compensation

11.8. Management of pregnancy after study enrollment

11.9. General medical care

11.10. HIV-related care

11.11. Medical care for study drug associated adverse event

11.12. Publication and presentation policy

12. Clinical safety management and reporting…………………………………………... ..31

12.1. Definitions

12.2. Adverse event

12.3. Adverse drug reaction

12.4. Unexpected adverse drug reaction

12.5. Serious adverse event

12.6. Evaluation of adverse events

12.7. Reporting of safety events

12.7.1. Expedited reporting

12.7.2. Routine periodic reporting

12.7.3. Documented on clinical forms but not otherwise reported

12.8. Clinical management of adverse events

12.8.1. Management of laboratory abnormality or clinical event

12.8.2. Management of serum creatinine elevation

13. Statistical summary………………………………………………………………….. 35

13.1. Safety outcome

13.2. Effectiveness outcome

13.3. Measures to minimize bias

13.4. Randomization

13.5. Blinding

13.6. Unblinding procedure

13.7. Analysis plan summary

13.8. Analysis of safety outcomes

13.9. Efficacy analysis

13.10. Interim analysis plan summary

14. Monitoring plan……………………………………………………………………… 38

14.1. Clinical monitoring plan

14.2. Safety monitoring plan

14.3. Protocol compliance monitoring plan

14.4. Emergency Protocol Violations

14.5. Non-emergency protocol violations

15. Data management plan………………………………………………………………. 40

15.1. Data management and analysis software

15.2. Quality assurance of form data collection

15.3. Data storage

16. Administrative procedures…………………………………………………………... 41

16.1. Study initiation

16.2. Study conduct

17. Study coordination…………………………………………………………………... 42

17.1. Study completion

17.2. Site record retention and access to documents at the site

18. Laboratory specimens and biohazard containment………………………………….. 43

18.1. Laboratory testing

18.2. Specimen storage and possible future research testing

18.3. Biohazard containment

19. References…………………………………………………………………………… 44

Appendices

A. Bangkok Tenofovir Study Screening Consent Form

B. Bangkok Tenofovir Study Enrollment Consent Form

C. Bangkok Tenofovir Study Laboratory Specimen Testing

D. Gilead Modified Common Toxicity Grading Scale – Adults

E. HIV testing algorithm

**1. Bangkok Tenofovir Study Group**

| **Bangkok Tenofovir Study Group** |  |
| --- | --- |
| Dr. Kachit Choopanya | Principal Investigator |
| **Bangkok Metropolitan Administration** |  |
| Dr. Kraichack Kaewnil  Deputy Permanent Secretary BMA | Investigator |
| **BMA, Department of Health** | Investigator |
| Dr. **Montira Thongsari**  Director General |  |
| Dr. Wantanee Wattana  Deputy Director General | Investigator |
| Dr. Parnrudee Manomaipiboon  Director, Drug Abuse Prevention and Treatment Division | Investigator |
| **BMA, Medical Services Department** |  |
| Dr. **Pirapong Saicheua**  Director General Department of Medical Services | Investigator |
| Dr. **Sravudthi Sonthikaew**  Deputy Director General Department of Medical Services | Investigator |
| **Clinical Coordination Team** |  |
| Dr. Suphak Vanichseni | Investigator, Clinic Coordinator |
| Dr. Udomsak Sangkum | Investigator |
| Dr. Pravan Suntharasamai | Investigator, Good Clinical Practices Coordinator |
| **Thailand Ministry of Public Health** |  |
| Dr. **Pachara Sirivongrangson**  Acting Director, Bureau of AIDS, TB, STIs | Investigator |
| Dr. Somyot Kittimunkong  Chief, AIDS Cluster | Investigator |
| **Thailand MOPH – U.S. CDC Collaboration** |  |
| Dr. Michael Malison, Director | Investigator |
| Dr. Pasakorn Akarasewi  Adjunct Director | Investigator |
| Dr. Frits van Griensven  Chief, Behavioral Science Section | Investigator and Senior Behavioral Scientist |
| Dr. Michael Martin  Chief, HIV Clinical Research Section | Investigator |
| Mr. Philip Mock  Chief, Data Management Section | Senior Data Manager |
| Dr. Janet McNicholl  Chief, Laboratory Sciences Section | Laboratory Coordinator |
| Dr. Robert Linkins  Director, HIV/STD Research Program | Investigator |
| Dr. Rutt Chuachoowong  Medical Scientist | Investigator |
| Anchalee Varangrat  Behavioral Scientist | Investigator |
| **United States Centers for Disease Control and Prevention** |  |
| Dr. Peter Kilmarx  Chief, Epidemiology Branch, Divisions of HIV/AIDS Prevention | Investigator |
| Dr. Lynn Paxton | Investigator |
| Dr. Michael Hendry  Chief, Laboratory Branch, Division of HIV/AIDS Prevention | Investigator |

1.1. Roles and Responsibilities

The Bangkok Tenofovir Study Group and Bangkok Metropolitan Administration

- Collaborate in protocol development and logistics of study procedures
- Submit protocol to Ethical Review Committees of the BMA, and the Thailand Ministry of Public Health
- Train clinic staff in study procedures and counseling
- Screen and obtain written informed consent from participants
- Enroll participants, provide study drug, and arrange follow-up
- Perform data and specimen collection and counseling
- Perform urine testing for pregnancy
- Evaluate, treat, and refer HIV infected study participants
- Provide secure, private clinic space for evaluation and counseling of participants.

*Thailand Ministry of Public Health – U.S. CDC Collaboration*

- Develop full protocol with input from collaborators
- Submit protocol to the United States CDC Institutional Review Board for approval
- Assist in training clinic staff in study procedures and counseling
- Provide study materials including questionnaires, study forms, office supplies, specimen collection materials and laboratory equipment
- Handle specimens and perform laboratory testing as defined in protocol
- Perform data management
- Monitor trial data and study staff performance
- Perform data analysis, interpretation, and communication of results with input from collaborators

*Thailand Ministry of Public Health*

- Collaborate in protocol development
- Facilitate communication with MOPH Ethical Review Committee
- Assist with data and laboratory analysis and interpretation and communication of results

*Division of HIV/AIDS Prevention-Surveillance and Epidemiology, Epidemiology Branch, Centers for Disease Control and Prevention*

- Collaborate in protocol development and logistics of study procedures
- Facilitate communication with CDC Institutional Review Board and CDC consultants
- Assist with development of data collection instruments, data and laboratory analysis and interpretation and communication of results
- Provide financial support to both BTSG and TUC to prepare for and to conduct the Bangkok Tenofovir study as described in the protocol

**2. Protocol Summary**

**Title:** Study of the Safety and Efficacy of Daily Tenofovir to Prevent HIV Infection Among Injection Drug Users in Bangkok, Thailand (Bangkok Tenofovir Study)

**Objectives:** Primary objectives:

- To determine if daily oral tenofovir 300 mg prevents HIV infection among injection drug users (IDUs) in Thailand
- To determine if daily oral tenofovir 300 mg is safe when given to HIV-uninfected IDUs in Thailand

Secondary objectives:

- To evaluate changes in HIV associated risk behaviors during the trial
- To evaluate adherence of IDUs to daily tenofovir/placebo
  - - To determine if daily tenofovir is associated with a lower HIV viral load set point or higher CD4 count among trial participants who become HIV infected
    - To determine if tenofovir is associated with the development of antiretroviral resistance among trial participants who become HIV infected
    - To determine if the genetic characteristics of HIV viruses that infect placebo recipients differ from HIV viruses that infect tenofovir recipients

**Design:** A phase II/III, randomized, double-blind, placebo-controlled study of the efficacy and safety of oral tenofovir (tenofovir disoproxil fumarate) 300 mg administered once daily to HIV-uninfected IDUs.

In phase II, IDUs will be randomized 1:1 to once daily tenofovir or placebo until 200 person-years of observation have accrued when a data safety monitoring board (DSMB) safety review will be done. If safety is confirmed, all phase II participants will continue, and additional participants will be enrolled into the phase III trial until 2,400 persons have been enrolled. Participants will choose between daily follow-up with directly observed therapy (DOT) or monthly follow-up. Participants will remain on assigned study medication until 40 endpoints have accrued.

**Population:** HIV-uninfected Thai IDUs ages 20-60 years.

**Study Sites:** The study will be conducted at 17 Bangkok Metropolitan Administration (BMA) drug treatment clinics located in Bangkok, Thailand.

**Duration:** Phase II/III is estimated to take 48 months; 36 months to enroll 2,400 participants, and an additional 12 months to complete follow-up.

**Test Products:** Tenofovir 300 mg or placebo.

**Study Procedures:** Interviews, risk reduction counseling, physical examinations, and/or laboratory evaluations at screening, enrollment, and every 4 weeks while on tenofovir or placebo.

**Endpoints:** HIV seroconversion, adverse events, rates of injecting and needle sharing, adherence to study drug/placebo, HIV viral load and CD4 counts, antiretroviral resistance, and genetic characteristics of infecting HIV viruses.

**Eligibility Criteria:** Potential participants must be age 20-60 years, HIV-uninfected, report injection drug use during the 12 months before trial enrollment, be capable of providing blood, oral secretions and urine, understand the protocol procedures, and provide written informed consent.

3**. Abbreviations and acronyms**

ACASI audio computer assisted self-interview

AE adverse event

AIDS acquired immunodeficiency syndrome

ALT (SGPT) alanine aminotransferase

ART antiretroviral therapy

AST (SGOT) aspartate aminotransferase

β-HCG β-human chorionic gonadotropin

BMA Bangkok Metropolitan Administration

BMD bone mineral density

BTSG Bangkok Tenofovir Study Group

BVEG Bangkok Vaccine Evaluation Group

CBC complete blood count

CD4 cluster of differentiation 4

CDC U.S. Centers for Disease Control and Prevention

CRC Community Relations Committee

CRF case report form

dL deciliter

DOT directly observed therapy

DSMB data safety monitoring board

EDTA Ethylene diamine tetra-acetic acid

EIA enzyme immunoassay

ERC Ethical Review Committee

Extension Study Extended follow-up of participants in the phase III trial of AIDSVAX B/E HIV vaccine

FDA Food and Drug Administration

g gram(s)

GCP Good Clinical Practice guidelines

HAART highly active antiretroviral therapy

HBV hepatitis B virus

HIV-1 human immunodeficiency virus, type 1

HPLC high performance liquid chromotography

ICH International Conference on Harmonization

IDUs Injection Drug Users

IRB Institutional Review Board

IU international unit(s)

kg kilogram(s)

LLN lower limit of normal

MCH maternal and child health

mg milligram(s)

mL milliliter(s)

mm^3^ cubic millimeter(s)

MOPH Thailand Ministry of Public Health

NNRTI non-nucleoside reverse transcriptase inhibitor

NRTI nucleoside reverse transcriptase inhibitor

NtRTI nucleotide reverse transcriptase inhibitor

PBMC peripheral blood mononuclear cells

PCR polymerase chain reaction

PEP post-exposure prophylaxis

PI protease inhibitor

po by mouth

PrEP pre-exposure prophylaxis

QA quality assurance

QC quality control

RT reverse transcriptase

RTI reverse transcriptase inhibitor

SAE serious adverse event

SIV simian immunodeficiency virus

SST serum separator tube

TDF Tenofovir Disoproxil Fumarate

TUC Thailand MOPH – U.S. CDC Collaboration

µg microgram(s)

ULN upper limit of normal

VAX003 Phase III trial of AIDSVAX B/E HIV vaccine

WB Western Blot

**4. Signature page**

**Title:** Study of Daily Tenofovir Disoproxil Fumarate to Prevent HIV Infection Among Injection Drug Users in Bangkok, Thailand (Bangkok Tenofovir Study)

**Sponsor:** Epidemiology Branch

Division of HIV/AIDS Prevention-Surveillance and Epidemiology

National Center for HIV, STD, and TB Prevention

U.S. Centers for Disease Control and Prevention (CDC)

1600 Clifton Road, Mailstop E-45

Atlanta, GA 30333, USA

I, the Principal Investigator, agree to conduct this study in full accordance with the provisions of this protocol. I also agree to maintain all study documentation for at least five years following study completion.

I have read and understand the information in the Investigator’s Brochures, including the potential risks and side effects of the products under investigation, and will ensure that all associates, colleagues, and employees assisting in the conduct of the study are informed about the obligations incurred by their contribution to the study.

Dr. Kachit Choopanya

__________________________________

Name of Investigator of Record

__________________________________ _________________________________

Signature of Investigator of Record Date

**5. Introduction**

**5.1 Project justification**

In Thailand, the HIV epidemic began among IDUs in 1988, with the prevalence rising from less than 1% to more than 40% in 1 year.^1^ As part of a national plan to develop and evaluate HIV vaccines,^2^ a cohort study of 1,209 IDUs was conducted from 1995–1998 in Bangkok.^3^ The study was conducted at 17 drug treatment clinics of the BMA. Each year, approximately 8,000-10,000 IDUs are treated at BMA drug treatment clinics. Treatment regimens include 45-day methadone detoxification and methadone maintenance.

Among IDUs screened for the cohort study, 29.9% were HIV-seropositive. The HIV incidence rate was 5.8 per 100 person-years; 88.2% of eligible participants returned for follow-up visits at 12 months, 75.9% at 24 months, and 71.2% at 36 months. The successful planning and conduct of this cohort study contributed important background data and research infrastructure that lead to the first phase III HIV vaccine trial in Asia. From March 1999 – August 2000, 4,943 IDUs were screened for enrollment in the AIDSVAX B/E HIV vaccine trial; their median age was 26 years, 94.3% were male, and 34.2% were HIV-seropositive.^4^ Among the 2,545 IDUs who enrolled in the vaccine trial, 93.8% reported injection drug use during the 6 months before enrollment. The frequency of drug use was high, with 39.4% reporting use daily and 32.7% at least weekly. The annualized HIV incidence rate during the 3-year trial (i.e., 1999 – 2003) was 3.4 per 100 person-years, varying from 2.1 to 4.2 during the course of the trial with no clear temporal trend (unpublished data).

During the vaccine trial, HIV education and risk-behavior counseling were provided at every study visit according to a standard operational protocol. Reports of injection drug use and needle sharing decreased significantly during the vaccine trial.^5^ The use of condoms and bleach to clean injection equipment were provided free of charge at every visit. Consistent with Thailand’s HIV prevention policy, drug injection equipment was not provided or exchanged at participating clinics, but sterile syringes and needles are readily available without prescription at pharmacies for 8 Thai baht (about US$0.20). Despite frequent, client-centered counseling, HIV incidence remained above 3% per year during the vaccine trial.

Based on population estimates^6^ and data from the AIDSVAX B/E vaccine trial, approximately 1,000 IDUs are infected with HIV each year in Bangkok. The number of candidate HIV vaccines in clinical trials has increased in recent years.^7^ Nonetheless, an effective preventive HIV vaccine is still years away and there is an urgent public health need to find other HIV prevention tools, particularly for those who are unable or unwilling to respond to currently available behavioral interventions. Tenofovir, a once-daily antiretroviral with low toxicity and slow development of resistance mutations, may be such a prevention tool.

**5.2 Why tenofovir?**

There is ample evidence in animals and humans that post-exposure prophylaxis, if given promptly, can reduce the risk of acquiring HIV infection. In the case of health care workers with needle-stick injuries from HIV-infected patients, zidovudine monotherapy is estimated to reduce the risk by 81%.^8^ It is biologically plausible and consistent with data from both perinatal HIV prevention studies and other infectious diseases that pre-exposure prophylaxis (PrEP) would be at least as effective and likely more so.

Suggested criteria for an ideal PrEP agent include the following:^9^

- Potent antiretroviral drug
- Rapidly bioavailable and long duration of action
- Easily administered on a once-daily or less dosing schedule
- Demonstrated low rates of clinical toxicity and hypersensitivity and well tolerated
- Favorable drug interaction profile for commonly used medications
- High “genetic barrier” to the emergence of drug resistance
- Affordable

Tenofovir meets all these criteria and in animal studies, it has shown the ability to protect non-human primates against intravenous, oral, and mucosal simian immunodeficiency virus (SIV) challenge.^10^ In addition, tenofovir is approved for use in treating persons with HIV infection in both the U.S. and Europe, has minimal drug-drug interactions, and few clinical contraindications.

**5.3 Why a phase II safety study?**

While multiple phase I and II studies have been done to assess the safety, tolerability, and pharmacokinetics of tenofovir for treatment of HIV infection, there are limited data on tenofovir use among HIV-uninfected persons. A study of 14 HIV uninfected subjects on stable doses of tenofovir showed that tenofovir 300 mg once daily did not affect the pharmacodynamics or pharmacokinetics of methadone.^11^ Nonetheless, additional safety data are needed to ensure tenofovir can be safely used by IDUs.

**5.4 Potential increase in HIV associated risk behavior**

In populations being studied for PrEP and other HIV prevention strategies (e.g., vaccines, microbicides), concerns have been raised about increased HIV associated risk behavior. It is possible that study participants will, despite education otherwise, believe that they are protected by the study medication and continue or increase risk behaviors. However, data available from previous studies in this IDU population suggest that HIV associated risk behaviors will decline.^3,5^ Active community participation will be sought to ensure appropriate risk reduction messages are presented to study participants.

**5.5 Tenofovir adherence**

HIV-infected participants in the AIDSVAX B/E HIV vaccine trial were offered ART. Data from the vaccine trial and published research suggest that IDUs in Bangkok will adhere to daily oral medications.^12,13^ Clinic staff will observe participants who choose daily follow-up take their daily dose of tenofovir (directly observed therapy – DOT). Active community participation will be sought to ensure appropriate adherence messages are presented to study participants. Adherence counseling will be refined based on input from the community.

**6. Study objectives**

**6.1 Primary objectives**

- To determine if daily oral tenofovir 300 mg prevents HIV infection among IDUs
- To determine if daily oral tenofovir 300 mg is safe when given to HIV-uninfected IDUs

**6.2 Secondary objectives**

- To evaluate changes in HIV associated risk behaviors during the trial
- To evaluate adherence of IDUs to daily tenofovir/placebo
  - - To determine if daily tenofovir is associated with a lower HIV-1 viral load set point or higher CD4 count among trial participants who become HIV infected
    - To determine if tenofovir is associated with the development of antiretroviral resistance among trial participants who become HIV infected
- To determine if the genetic characteristics of HIV viruses that infect placebo recipients differ from HIV viruses that infect tenofovir recipients

**7. Trial design**

This is a phase II/III, randomized, double-blind, placebo-controlled study of the safety and efficacy of chemoprophylactic tenofovir, administered orally once daily to IDUs. The study will be conducted in Bangkok at 17 BMA Drug Treatment Clinics. Study participants will be randomized (1:1) to receive tenofovir 300 mg or placebo. Participants will be evaluated for adverse events and HIV seroconversion.

The primary goals of this study are to assess the safety and efficacy of daily tenofovir to prevent parenteral HIV infection among IDUs. Assessment of changes in HIV associated risk behaviors, adherence to study drug, and, among IDU who become HIV-infected during the trial, evaluation of HIV viral load set point, CD4 counts, genetic characterization of infecting HIV viruses, and antiretroviral resistance will also be done.

**7.1 Primary endpoints**

The primary efficacy endpoint will be measured by:

- Rates of HIV seroconversion measured at monthly intervals

The primary safety endpoints will be measured by:

- The frequency of Grade 3 or 4 renal or hepatic function laboratory toxicities or clinical toxicities in blinded tenofovir and placebo arms, as defined by the Gilead-modified NIAID Adult Common Toxicity Tables, and which cannot be directly attributed to a cause other than study medications
- The frequency of adverse clinical events in tenofovir and placebo arms

**7.2 Secondary endpoints**

Changes in HIV associated risk behaviors will be measured by:

- Rates of reported injection drug use and injection drug use frequency during the trial
- Rates of reported needle sharing
- The number of unprotected sexual acts over the course of the trial
- Number of reported sexual partners over the course of the trial
- Proportional use of condoms during sexual intercourse

Medication adherence will be measured as:

- Rates, by interview and documentation on tenofovir adherence card, of participants taking at least six (86%) of seven daily doses of study drug each of the four weeks preceding the monthly study visit

Differences in virologic and immunologic responses to HIV infection among tenofovir and placebo recipients will be measured by:

- Plasma viral load, measured by quantitative RNA PCR, a predictor of clinical progression of HIV disease;^14^ CD4 cell counts will be measured by flow cytometry
- Rates and nature of HIV antiretroviral genotypic and phenotypic resistance will be measured
- Genetic characteristics of infecting HIV viruses including DNA sequence analysis and antibody binding studies will be conducted

**8. Study Design**

In phase II, participants will be followed months 0, 1, 2, 3, then 3 monthly with hematology and chemistry tests and laboratory evaluations of renal and hepatic function until 200 person-years of observation are accrued. At that point, a DSMB safety assessment will be conducted. Follow-up of enrolled participants will continue during the DSMB safety assessment. If safety is confirmed, all phase II participants will continue, and additional participants will be enrolled into the phase III portion of the trial. Enrollment of 2400 participants or accrual of 24 HIV infections (whichever comes first) is anticipated to take 36 months.

Participants will choose between two follow-up schedules: monthly (every 4 weeks) or monthly plus daily with directly observed therapy (DOT). During DOT visits clinic staff will witness the participant swallow his/her study medication and clinic staff will initial the participant’s tenofovir adherence card. Monthly visits will be the same for both groups and will include an assessment of tenofovir adherence and adverse events, a pill count and collection of unused pills, provision of a new 1 month supply of study medication, pre- and post-test HIV counseling, rapid oral HIV testing, urine pregnancy test (for female participants), HIV risk reduction counseling, and medication adherence counseling. At 0, 3, then 3 monthly visits, monthly procedures will be supplemented with a risk behavior questionnaire.

**8.1 Study population**

The population to be included in this study is IDUs in Bangkok.

**8.1.1 Recruitment**

IDUs seeking treatment at BMA drug treatment clinics will be offered HIV counseling and testing and screening for the tenofovir study. Subjects may be recruited from existing IDU cohorts (e.g., the AIDSVAX B/E Extension Study [CDC #3750]). In addition, an IDU peer referral program will be conducted.

**8.2 Inclusion criteria**

- 20-60 years of age
- Report injection drug use in the 12 months before screening
- Possess documentation of Thai National Identification number
- Laboratory values as follows within 2 weeks before enrollment:
  - HIV oral fluid test non-reactive at screening and pre-enrollment visits
  - Hemoglobin  9 gm/dL
  - ALT and AST  2.5 x upper limit of normal (ULN)
  - Total bilirubin  1.5 mg/dL
  - Serum amylase  1.5 x ULN
  - Serum phosphorus 2.2 mg/dL
  - No evidence of current or chronic Hepatitis B infection by serology
  - Calculated creatinine clearance 60 mL/min by the Cockcroft-Gault formula where creatinine clearance in mL/min =

Male: (140 – age in years) x (wt in kg)

72 x (serum creatinine in mg/dL)

Female: (140 – age in years) x (wt in kg) _x 0.85_

72 x (serum creatinine in mg/dL)

- Willing to abstain from sexual intercourse or use effective contraception during the trial (oral, injection, or barrier) (women)
- Pass the comprehension test
- Willing and able to provide informed consent for study participation
- Available and committed to DOT or monthly follow-up for at least 12 months

**8.3 Exclusion criteria**

- - Clinic physicians will determine if a subject with chronic illness requiring prescription medication can not enroll (medication used for drug treatment is allowed)
  - Positive urine pregnancy test (women)
  - Breastfeeding (women)
  - History of significant renal, liver, or bone disease
  - Any other clinical condition or prior therapy that, in the opinion of the clinic physician, would make the subject unsuitable for the study or unable to comply with the dosing requirements
  - Concurrent participation in any other HIV prevention trial or drug/vaccine safety trial. AIDSVAX B/E HIV vaccine trial (CDC protocol #2076) participants and Extension Study (CDC protocol #3750) participants may be screened for enrollment in the Bangkok Tenofovir Study.

**8.4 Participant retention**

Study staff will make every effort to retain participants for the duration of the trial to minimize possible bias associated with participant loss-to-follow-up. Components of retention procedures will include:

- A comprehension test administered to each potential participant to assess their understanding of the trial; subjects must pass the test in order to enroll
- A thorough explanation of the study visit schedule and procedures during the informed consent process and re-emphasis at each study visit
- Collection of locator information during screening/enrollment and at each study visit
- Use of appropriate and timely visit reminder mechanisms (e.g., appointment calendar, phone, post cards)
- Follow-up of missed visits by clinic staff as described in the informed consent (e.g., phone call, post card, home visit)
- BMA will coordinate with appropriate authorities (e.g., Ministry of Justice, Police Department) to allow follow-up of incarcerated study participants

**8.5 Participant withdrawal**

Participants may withdraw from the study for any reason at any time. The Principal Investigator also may withdraw participants from the study to protect the participant’s health and/or if the participant is unwilling or unable to comply with required study procedures. Such reasons include, but are not limited to, grade 4 creatinine elevation or grade 3 with recurrence, pregnancy, or the onset of health conditions that compromise study participation. Participants may also be withdrawn if the study sponsor, the BMA, the Royal Thai Government, or regulatory authorities terminate the study prior to its planned end date.

**8.6 Community relations committee**

In preparation for the AIDSVAX B/E vaccine trial, BMA staff and IDUs established a Community Relations Committee (CRC).^4^ The CRC was formed in order to establish mechanisms to solicit and respond to community attitudes about study procedures, the reputation of the study in the community, and questions arising from participants or the general community. The BTSG and BMA staff will work with IDU community representatives to support and continue the work of the CRC. The CRC will be composed of IDUs, their family members, and representatives of local organizations and community sectors. The CRC will meet every 2 months.

The CRC will provide a forum for IDUs to express their views, clarify their needs, and contribute to the planning and conduct of the tenofovir study. During these meetings, the background, purpose, procedures, and measures taken to ensure participant confidentiality and privacy will be explained and discussed. Furthermore, the risks of participation, the voluntary nature of study participation, and informed consent will be reviewed. The outcomes of these discussions will be used to adjust and guide the execution of the study and to raise community involvement and support for the project.

**9. Study products**

Two study products will be used during the trial, tenofovir and placebo. Each tablet of tenofovir and placebo is film-coated to mask taste with lactose monohydrate, hydroxypropyl methylcellulose, titanium dioxide, triacetin, and FD&C Blue No. 2 aluminum lake for color.

Each product will be provided by Gilead Sciences, Inc. in white, 60 mL, high-density polyethylene bottles with white child-resistant caps. Each bottle will contain 30 tablets as well as a single silica gel canister to protect the product from humidity and fiber packing to protect the product during handling and shipping. Each bottle will be labeled with a randomization number, the protocol number, and expiration date.

**9.1 Tenofovir disoproxil fumarate (Tenofovir)**

**9.1.1 Chemical and pharmacokinetic properties**

Each tenofovir tablet provided by Gilead Sciences, Inc. contains 300 mg of tenofovir and the following inactive ingredients: microcrystalline cellulose, lactose monohydrate, pregelatinized starch, crocarmellose sodium, and magnesium stearate.

Tenofovir Disoproxil Fumarate (tenofovir), 9-[I-2-[[bis[[isopropoxycarbonyl)oxy] methoxy]phosphinyl]methoxy]propyl] adenine fumarate, is the oral pro-drug of the intravenous compound 9-[I-2-(phosphonomethoxy) propyl] adenine monohydrate. Tenofovir inhibits the activity of HIV reverse transcriptase by competing with the natural substrate deoxyadenosine 5’-triphosphate and, after incorporation into DNA, by DNA chain termination.

Tenofovir is rapidly absorbed with peak serum concentration reached between 0.5 and 1.5 hrs after oral dosing. Tenofovir is eliminated primarily by renal excretion and has an estimated terminal half-life of approximately 6-8 hours. In non-pregnant adults, the intracellular half-life for the disappearance of tenofovir, the active metabolite, is 12 to 15 hours in activated lymphocytes and 33 to 50 hours in resting lymphocytes.

Based on laboratory data using the HIV-1 IIIB strain in PBMCs, the concentration that inhibits 50% (IC50) of viral isolates is 0.18 µM or ~52 ng/mL. Equivalent antiviral activity has been shown against HIV-1 clade C and other HIV-1 non-B clade primary isolates (A, C, D, E, F, G, and group O).^15^

**9.1.2 Clinical trials**

The U.S. FDA approved Viread® (tenofovir) in October 2001 for use in combination with other antiretroviral therapeutic drugs for the treatment of HIV infection. Over 12,000 patients have participated in clinical trials or expanded access studies, and tenofovir has been shown to be generally safe, effective, and well tolerated. Ongoing clinical studies are examining tenofovir for use in new populations for new indications. Three studies are being conducted among HIV-infected pediatric populations to examine a suspension formulation. Other studies are assessing safety among adults with hepatitis B infection and for use as an intravaginal topical gel.

**9.1.3 Safety profile**

Tenofovir has almost exclusively been studied in HIV-infected adults, either as monotherapy for brief periods to assess safety or pharmacokinetics, or as a component of multi-drug combination therapy for treatment of HIV disease. The concomitant use of multiple antiretrovirals does not allow determination of the rate of toxicities we can expect when tenofovir is administered alone. However, data from placebo-controlled studies are available to examine the added risk of tenofovir in multidrug combination therapy.

Changes in bone growth and strength have been seen in study animals given tenofovir. It is unknown if tenofovir will cause bone abnormalities in humans. In studies of pregnant macaques given very high doses of tenofovir during pregnancy (6-12 times the dose to be used in this study), there was a reduction in bone mineralization in the macaque infants.^16^ However, in human tenofovir trials (in HIV-infected persons) to date, fracture rates have been lower in the tenofovir arm than in the placebo arm and nearly all fractures resulted from trauma.^10^ All reported bone fractures will be recorded as AEs, or if appropriate, SAEs and analyzed at interim and final DSMB safety analyses.

In a escalating dose study of tenofovir among 49 subjects, there were five grade III or IV adverse events deemed possibly or probably related to the study agent among 38 subjects who received tenofovir,^18^ and an equivalent proportion (1 of 11) of participants receiving placebo. The adverse events consisted of elevated serum creatinine kinase (n=5) and elevated liver transaminases (n=2). One tenofovir recipient developed peripheral neuropathy; this adverse event also occurred in one placebo recipient. All events resolved completely after discontinuation of the drug or placebo.

In a phase III, randomized, 600 antiretroviral-naïve participant, double-blind study of tenofovir + lamivudine + efavirenz compared to stavudine + lamivudine + efavirenz (903) the incidence of grade 3 or 4 adverse events and laboratory abnormalities was similar between the two groups.^10^ Through 96 weeks, nucleoside-related toxicities (e.g., peripheral neuropathy, pancreatitis, and lactic acidosis) were significantly lower in the tenofovir arm. Another phase III, randomized, double-blind trial (907) with 550 treatment-experienced participants, the incidence of clinical or laboratory adverse events was similar in tenofovir and placebo arms. (Table 1)

**Table 1. Study 907. Percentage of patients who experienced grade 3 or 4 events (occurring in >2 patients) regardless of relationship to study drug.**

| **Type of Event** | **Placebo**  **(N=182)**  **(Week 0-24)** | **Tenofovir**  **(N=368)**  **(Week 0-24)** |
| --- | --- | --- |
| **Clinical Adverse Event**  **Grade 3 or 4*** | | |
| Asthenia | <1 % | <1 % |
| Pain | <1 % | <1 % |
| Myalgia | 0 | <1 % |
| Abdominal pain | <1 % | <1 % |
| Nausea | 1 % | <1 % |
| Diarrhea | 2 % | <1 % |
| Pancreatitis | 0 | <1 % |
| Bacterial Infection | <1 % | <1 % |
| Cellulitis | <1 % | <1 % |
| Pneumonia | 0 | <1 % |
| Sinusitis | 1 % | <1 % |
| Renal Calculus | 0 | <1 % |
| Anxiety | 0 | <1 % |
| Depression | <1 % | <1 % |
| *Events causing permanent study drug discontinuation* | 2 % | 3 % |
| **Laboratory Abnormalities**  **Grade 3 or 4*** | | |
| Triglycerides | 13 % | 8 % |
| Creatine Kinase | 14 % | 7 % |
| Amylase | 7 % | 6 % |
| Urine Glucose | 3 % | 3 % |
| Serum Glucose | 4 % | 2 % |
| AST | 3 % | 3 % |
| ALT | 2 % | 2 % |
| Neutrophils | 1 % | <1 % |
| *Events causing permanent study drug discontinuation* | 2 % | 0 |

*see Appendix D for severity grading

Of special concern for the IDU population is the possibility of a drug-drug interaction between tenofovir and methadone. The pharmacodymamics (PD) and pharmacokinetics (PK) of tenofovir were examined in a study among 14 HIV-uninfected IDUs on stable doses of methadone. Following 14 days of directly observed therapy methadone PD and PK exposures were similar among tenofovir and placebo recipients.^11^

As of 30 April 2003, approximately 153,000 persons have taken tenofovir in clinical studies, expanded access programs, and in postmarketing settings in the United States, Europe, and Australia. Tenofovir has been generally well tolerated with few adverse events. It is reasonable to hypothesize that side effects and toxicities will be less in healthy, HIV-uninfected persons.

**9.2 Placebo**

Each placebo tablet provided by Gilead Sciences, Inc. contains the inactive ingredients listed above and denatonium benzoate to provide a bitter taste to match the tenofovir tablets.

**9.3 Drug accountability**

The study site~~s~~ will periodically receive a supply of study products, labeled with the randomization code. Study product will be stored in a limited access area that is securely locked and temperature controlled (range of 15° C – 30° C).

It has been demonstrated that tenofovir disoproxil fumarate tablets are stable at 25 degrees C and 60% relative humidity for up to 36 months; at 30 degrees C and 65% relative humidity for 18 months; and at 40 degrees C and 75% relative humidity for 6 months. Although tenofovir disoproxil fumarate tablets and placebo to match tenofovir disoproxil fumarate tablets should be stored at the clinical site(s) under controlled temperature and humidity conditions (25 degrees C; excursions not exceeding 15 degrees C to 30 degrees C) to ensure stability of the product, the aforementioned data does support dispensing a 30 day supply to study subjects who may keep their study drug in conditions that exceed these recommended storage conditions.

Strict inventory will be maintained on all study product received. Study product that has expired or been returned unused by participants will be destroyed as clinical waste in Thailand by incineration or burial.

**9.4 Adherence counseling**

Adherence counseling will be based on adherence research conducted among IDUs.^13,19,20^ The counseling session will be individualized and include the following:

- Basic education about study medications (e.g., dose and schedule, how tenofovir works, side effects and how to handle them, refills and storage, do’s and don’ts of taking study medication, etc.)
- Discussion of the participant’s beliefs and attitudes about taking tenofovir/placebo (e.g., concerns, expectations, fears)
- Encouragement about talking to study personnel about medications (e.g., what to tell study personnel about your medications, questions you might have and should feel free to ask, the need to be open even when adherence is poor so study procedures and adherence counseling can be improved)
- Identifying and solving barriers to adherence (e.g., scheduling and organizational skills, reminder devices, social support, disclosure)
- Discussion about reasons not to engage in pill-sharing and how to handle requests from friends and family to borrow doses

At each follow-up visit, study counselors will review the study participant’s self-report of adherence, discuss barriers to adhering to the daily dose of study medication, and help identify strategies for overcoming barriers.

**9.5 Adherence monitoring**

We will best be able to assess safety and efficacy of tenofovir in light of knowledge about medication use, so monitoring adherence is important. In addition to direct observed therapy for a subset of trial participants, four methods will be used to assess adherence:

- Self-report on interview: To encourage reporting of nonadherence, participants will be reminded that we understand taking all doses is difficult and that we need them to tell us about missed doses
- Pill counts: At each monthly study visit
- Medication diary: At each monthly study visit diaries will be reviewed and compared to pill count
- Tenofovir blood levels will be determined on unblinded specimens at the conclusion of phase III from:
  - All HIV seroconverters – serum from the first HIV positive visit and the last HIV negative visit for tenofovir recipients. Results will be compared to diaries and pill counts to assess adequacy of self-report to predict therapeutic blood levels around the time of seroconversion.
  - Specimens from 10% of all persons who received tenofovir and did not seroconvert will be randomly selected from the first quarterly visit and the quarterly visit before going off-drug. Results will be compared to diaries and pill counts to assess correspondence to self-reported adherence.

**9.6 Study drug interruption**

The study drug may be temporarily interrupted if the participant runs out of study drug because of a missed visit or the physician stops study drug to allow resolution of a transient adverse event. In such cases, an HIV test will be done to confirm the participant is HIV-uninfected before the participant resumes study drug.

**9.7 Concomitant medications**

All concomitant medications will be reported on the appropriate case report form. In addition to prescribed and over-the-counter medications, methadone, vitamins, herbal remedies, and other ingested or inhaled traditional preparations will be recorded. Medications used for the treatment of adverse events (AEs) that occur during study participation will also be recorded on applicable study case report forms.

**10. Study visit summary (see Table 2 for details of each visit)**

**10.1 Screening visit**

At the screening visit, study staff will provide potential participants introductory information about the study. Those agreeing to participate will be given a screening ID number and a screening interview will be conducted. Study staff will provide a full description of the trial and procedures. Those who complete the screening and meet initial eligibility criteria will be given an appointment to review laboratory test results and final eligibility criteria.

**10.2 Enrollment visit**

Study staff will review inclusion and exclusion criteria with potential participants to determine trial eligibility. Those eligible will be assigned an enrollment ID number and appointments for the duration of study will be scheduled. Hepatitis B vaccination series will be initiated for hepatitis B seronegative participants.

**10.3 Follow-up visits**

Frequent follow-up visits will be conducted in order to:

1. Encourage adherence
2. Monitor symptoms, toxicities, and adverse events
3. Determine when HIV infection occurred as precisely as possible
4. Reduce the duration of monotherapy for those who become HIV-infected to limit development of antiretroviral drug resistance
5. Reduce recall bias in measures of adherence and HIV associated risk behavior

**10.3.1 Daily directly observed therapy (DOT)**

Participants will come to clinic each day and clinic staff will observe the participant swallow one tablet of tenofovir or placebo. Clinic staff will complete and sign the participant’s adherence card.

**10.3.2 Monthly visits (scheduled at 4 week intervals)**

Study staff will provide counseling, review study drug adherence, assess and record adverse event/s, do HIV and pregnancy tests, and collect/count unused medication and dispense a new supply. Participants will complete an adherence assessment questionnaire using an audio computer assisted self interview (ACASI).

**10.3.3 First two monthly visits and three monthly visits (weeks 4, 8, 12, 24, 36, etc.)**

Clinic staff will conduct all the monthly procedures. In addition, blood will be collected for laboratory testing. Participants will complete a risk behavior questionnaire using an ACASI at weeks 12, 24, 36, etc.

**10.3.4 Exit visit (Study completion, HIV infection confirmation visit, early termination)**

Clinic staff will conduct all the monthly and 3-monthly procedures. In addition, a blood specimen will be collected and a physical examination done.

**10.3.5 Follow-up visits for newly HIV infected participants**

All newly HIV infected participants will be followed at 4 weeks, 8 weeks, and every 16 weeks (4 months) after confirmation of HIV infection until the study ends. Participants will receive counseling, have a pregnancy test (women), and blood will be collected for laboratory testing. Participants will complete a risk behavior questionnaire using an ACASI at the 16 week (4 month) visits.

## Table 2. Bangkok Tenofovir Study Procedures Flow Chart

| **Procedures** | **Screening** | **Enrollment** | **Daily visit** | **Monthly visit** | **3 monthly visit** | **Exit visit** | **Post-analysis**  **monthly visits** | **Post-analysis**  **3-monthly visits** |
| --- | --- | --- | --- | --- | --- | --- | --- | --- |
| Screening informed consent | X |  |  |  |  |  |  |  |
| Screening questionnaire | X |  |  |  |  |  |  |  |
| Comprehension test |  | X |  |  |  |  |  |  |
| Physical exam^1^ |  | X |  |  |  | X |  |  |
| Enrollment eligibility |  | X |  |  |  |  |  |  |
| Enrollment informed consent |  | X |  |  |  |  |  |  |
| HIV pre/post-test counseling | X | X |  | X | X | X | X | X |
| OraQuick HIV testing | X | X |  | X | X | X | X | X |
| Urine pregnancy test (females) | X | X |  | X | X | X | X | X |
| HBV serology | X |  |  |  |  |  |  |  |
| Collect blood | X |  |  | M1 and M2 | X | X |  | X |
| Pill Count |  |  |  | X | X | X | X | X |
| Dispense 30 day supply of study medication |  | X |  | X | X |  |  |  |
| Review adherence diary |  |  | X | X | X | X |  |  |
| Adherence questionnaire (ACASI) |  |  |  | X | X | X |  |  |
| Adherence counseling |  | X |  | X | X | X | X | X |
| Risk assessment questionnaire (ACASI) |  | X |  |  | X | X |  | X |
| Risk reduction counseling | X | X |  | X | X | X | X | X |
| Update locator information | X | X |  | X | X | X |  |  |

1. A complete physical exam will be completed at enrollment and exit visits; a focused physical exam for specific participant complaints will be completed at other visits.

**10.3.6 Unscheduled visits**

Participants will be instructed to return to the clinic (without an appointment) as soon as possible if they experience symptoms or illness that may be related to study medication, upon release from a correctional facility if they have missed an appointment, if they no longer have access to study medication, or are unable to take study medication.

**10.3.7 HIV and additional testing in scheduled and exit visits**

If OraQuick test of oral fluid is non-reactive, the participant will be informed that there is no evidence of HIV infection. In order to ensure that daily oral tenofovir does not lead to OraQuick non-reactive test results among HIV-infected volunteers, HIV confirmatory nucleic acid testing (NAT) will be done on sera available from volunteers who test OraQuick non-reactive.

If OraQuick HIV testing is reactive, study medication will be stopped, and the participant will be informed that the test is reactive and that blood tests are required to confirm the result. If the participant agrees**,** blood will be drawn and confirmatory testing done (i.e., EIA and, if necessary Western Blot) and the participant will complete the risk assessment and adherence questionnaire.

At the follow-up visit in 2 weeks, participants will receive post-test counseling and results of confirmatory testing. Participants, who test positive on confirmatory HIV testing, will have HIV-1 viral load, HIV-1 genetic characterization, antiretroviral drug resistance and CD4 testing and will be referred for follow-up care. Participant specimens from previous visits will be tested to determine if the participant was in the HIV window period when they previously tested HIV negative by oral fluid testing.

**10.3.8. Follow-up visits if tenofovir safety and efficacy demonstrated**

If this study, or other tenofovir studies, determines that tenofovir is safe and effective in preventing HIV infection, HIV seronegative participants will be provided with tenofovir for at least 1 year. BMA clinic staff will be available to provide tenofovir daily to participants.

Monthly visits will include pre- and post-test HIV counseling, HIV risk reduction counseling, a rapid oral HIV test and HIV confirmatory testing if needed, and urine pregnancy test (for female participants). Visits at months 3, 6, 9, and 12 will include all monthly procedures plus a risk behavior questionnaire using ACASI and blood will be collected for laboratory safety testing.

**11. Ethics**

The study will be conducted in accordance with the Declaration of Helsinki, the Code of Federal Regulations, Title 45, Part 46 (45 CFR §46), and local ethical and legal requirements.

The protocol, including informed consent (Thai and English), and other requested documents, and any subsequent modifications, will be reviewed and approved by the ethical review committees responsible for oversight:

- Bangkok Metropolitan Administration
- Thailand Ministry of Public Health
- United States Centers for Disease Control and Prevention

After initial approval, all IRB/ERCs will review the protocol at least annually. The Principal Investigators will provide yearly trial updates to all IRB/ERCs including: the total number of participants enrolled, the number of participants who completed the study, and a line listing of all AE and SAE reports. All Data Safety and Monitoring Board (DSMB) reports will be provided to the IRB/ERCs. In addition, the Principal Investigator will provide a final report of trial results to the IRB/ERCs within three months of study termination or completion.

**11.1 Risks**

There are few anticipated health risks to participation in the trial other than minor discomfort associated with physical examinations and blood specimen collection. Some injection and sexual behavior questions may be embarrassing. There have been reports of decreased bone growth and bone strength in studies of tenofovir in animals and reports of renal injury in humans (please, see section 9.1.3).^10, 21-23^ A small number of persons randomized to tenofovir may experience changes is bone growth or renal function, but most will not and some persons randomized to placebo may also experience these health events. Participants will be monitored closely for clinical or laboratory evidence of bone or renal abnormalities. Based on previous studies, participants randomized to tenofovir may experience gastrointestinal discomfort (e.g., abdominal pain, nausea, vomiting, diarrhea). It is anticipated that prompt discontinuation of study product should quickly resolve any side effects and that no long-term effects should be experienced by the participants.

Among trial participants who become HIV-infected while on tenofovir there is a risk the virus will develop tenofovir resistance. Studies have shown that tenofovir resistance is uncommon, slow to develop, and low level.^10^ HIV tests will be performed monthly to limit time HIV infected participants will be on tenofovir monotherapy. Based on anticipated enrollment, expected HIV incidence, and trial data,^10^ it is unlikely any participants will develop tenofovir resistance. HIV infected participants will be tested for tenofovir resistance.

Participants may believe that study drug will protect them from HIV infection and may increase their HIV-related risk behaviors. Potential participants will be told during the informed consent process that we do not know if tenofovir prevents HIV infection and we will not know who is taking tenofovir. Risk reduction counseling will be provided at enrollment and at monthly follow-up visits (please see section 11.4.3). Analysis of data from the AIDSVAX B/E vaccine trial conducted in the same 17 drug treatment clinics showed that participant HIV-related risk behaviors declined by > 30% during the course of the 36 month study.^5^

Although study sites will make every effort to protect participant privacy and confidentiality, it is possible that participants’ involvement in the study could become known to others. Participants could face the risk of social harms, such as discrimination resulting from community perception that the individual is at high risk or already infected with HIV. Such discrimination may result in problems such as denial of insurance or employment. These risks will be minimized via thorough education, informed consent, and counseling to give subjects realistic expectations of an experimental medication and to emphasize avoidance of risk behaviors. We will collect details of social harm reports on a social harm report form and will treat reports of social harms as adverse events if they meet clinical AE criteria.

**11.2 Benefits**

Participants in this study will benefit from HIV education and risk reduction counseling and HIV testing. Methadone treatment, contraception, bleach (for cleaning injection equipment), and hepatitis B vaccination will be offered free of charge. Participants and others may benefit in the future from any information learned from this study that may lead to the development of a safe and effective intervention that prevents HIV infection.

**11.3 Post-trial access to effective products**

If this study, or other tenofovir studies, determines that tenofovir is safe and effective in preventing HIV infection, all HIV seronegative participants in this trial (both arms) will be provided with tenofovir at no cost for at least 1 year after trial termination. Tenofovir will be provided by Gilead Sciences, Inc. or purchased at cost by CDC.

**11.4 Counseling**

11.4.1 Pre-test counseling

Pre-test counseling will include an explanation of HIV infection and transmission, the meaning of HIV test results, risks associated with injection drug use, and an explanation of how to use condoms and how to use bleach to clean needles. Counseling messages will be tailored to each participant’s individual risk profile based on their sexual and drug use behaviors. Counselors will emphasize the unknown efficacy of tenofovir to prevent HIV infection and the double-blind, placebo controlled nature of the trial. Trained social workers and/or psychologists will perform all counseling.

11.4.2 Post-test counseling

Counseling will include goals, means, and strategies for behavioral risk reduction, maintenance of risk reduction, and explanation of risk reduction methods (e.g., condom use, cessation of drug use, cessation of needle sharing). Bleach and condoms will be provided free of charge.

Post-test counseling of HIV-infected participants will include an assessment of psychosocial needs, a discussion of living with HIV-infection and treatment, and issues related to discrimination. HIV transmission to partners will be discussed and strategies for behavioral change will be addressed (e.g., condom use, cessation of drug use, cessation of needle sharing).

**11.4.3 HIV risk reduction counseling**

All participants will receive HIV risk reduction counseling by trained study counselors at enrollment and each monthly visit. Trial participants will be informed of the risks associated with needle sharing, instructed on how to use bleach to clean needles, and provided with bleach free of charge. Participants will receive detailed information about methadone treatment programs available at the clinic. Participants will also be informed about sexual transmission of HIV, instructed in the use of condoms, and provided condoms free of charge. Participants will have an opportunity to ask questions about HIV infection and transmission at each visit.

**11.5 Informed consent**

Screening and enrollment informed consent forms were written in English then translated to Thai. In order to verify the translation, a translator not involved in the study, back translated the informed consents from Thai to English. Trained study staff will explain study procedures to the participants and discuss the advantages and disadvantages of participating. Staff will emphasize the voluntary and confidential character of the study, possible risks and benefits, alternatives to participation, and the participant’s right to withdraw from the study at any time.

**11.6 Participant confidentiality**

All study-related information will be treated as confidential. Study staff will be trained in procedures for maintaining participant confidentiality. A unique personal identification code will be used to identify questionnaires, laboratory specimens, and other materials. Records that contain names or other personal identifiers, such as locator forms and informed consent forms, will be stored in locked cabinets in areas with limited access. Electronic databases will be secured with password-protected access systems.

Participant’s study information will not be released without the written permission of the participant, except as necessary for study monitoring by: the BMA, the Thailand MOPH, the study monitor, the Thai FDA, Gilead Sciences, Inc., the U.S. CDC, the U.S. FDA, and/or other government and regulatory authorities.

**11.7 Compensation**

Participants will be compensated for their time, effort, and travel. Participants will receive 350 baht (~8.75) for each monthly study visit. Participants on the DOT schedule who come all 7 days in a week will receive 350 baht for that week. In addition, participants who return to clinic for DOT will be reimbursed 70 baht for that visit. If tenofovir safety and efficacy is demonstrated, participants choosing to take tenofovir will receive 350 baht for 3-monthly visit but will not be compensated for daily or monthly visits.

**11.8 Management of pregnancy after study enrollment**

Women who become pregnant during the study will stop taking study drug and be withdrawn from the study. Prenatal care will not be provided by the study, but women will be assisted with antenatal care arrangements. In addition, women who become pregnant will be offered immediate voluntary HIV counseling and testing. Women testing positive for HIV will be referred and encouraged to participate in BMA ‘prevention of mother to child transmission’ services. After obtaining participant consent, clinic staff will review the medical record of delivery to assess maternal and newborn health. Pregnancy data and pregnancy outcomes will be reported to the Antiretroviral Pregnancy Registry (tel: 1 910 256 0238, fax: 1 910 256 0637, +44 1628 789 666).

**11.9 General medical care**

The government of Thailand has a 30 baht (U.S. $0.75) program for emergency and general medical care. Patients pay 30 baht for each medical clinic or hospital visit. If a participant requires general medical care during the trial (e.g., broken arm, suture of laceration, pharyngitis), the sponsor will reimburse the participant the 30 baht cost of each medical visit.

**11.10 HIV-related care**

Thailand is expanding the government supported program to provide highly active antiretroviral therapy (HAART) and prophylaxis for opportunistic infections to all its citizens with a CD4 cell count < 200, an AIDS-defining illness, or prior antiretroviral therapy (ART). Trial participants who become HIV infected during the study will be referred to receive ART and prophylaxis for opportunistic infections (i.e., Standard Care) as described in the current *MOPH National Guidelines for Clinical Care of HIV-infected Adults and Children. 8^th^ e*d. The sponsor will assure medical care for HIV-infected participants until these participants are offered Standard Care.

**11.11 Medical care for study drug associated adverse event**

Evaluation of adverse events will be provided with treatment if indicated and available at the study clinic. If not available, referral to an appropriate provider will be arranged. If a severe adverse event is caused by the study drug, the sponsor will ensure the participant receives appropriate medical care.

**11.12 Publication and presentation policy**

Publication of the results of this study will be governed by BTSG. Presentations, abstracts, and manuscripts will be submitted for review by BTSG, TUC, CDC, and Gilead Sciences, Inc., prior to submission.

**12. Clinical safety management and reporting**

**12.1 Definitions**

For the purposes of documentation and reporting of events related to the safety of participants receiving study medications in these trials, the International Conference on Harmonization (ICH) definitions and terminology will be used.

**12.2 Adverse event (AE)**

An AE is any unfavorable and unintended sign (e.g., an abnormal laboratory finding), symptom, or disease temporally associated with the use of an investigational product, whether or not considered related to the product.

An AE **does not** include:

- Diseases or conditions present or detected prior to taking study drug (i.e., pre-existing) which do not worsen during the trial
- Medical or surgical procedures (e.g., endoscopy, appendectomy); but the condition that leads to the procedure is an AE (e.g., bloody diarrhea, appendicitis)
- Situations where an untoward medical event has not occurred (e.g., hospitalization for elective procedure or for social reasons/convenience)
- Overdose of either study drug or concomitant medication without any signs, symptoms, or hospitalization

**12.3 Adverse drug reaction (ADR)**

An ADR is an AE where a causal relationship between the study medication (taken in doses normally used) and the adverse event is at least a reasonable possibility.

**12.4 Unexpected adverse drug reaction (ADR)**

An unexpected ADR is an ADR, the nature or severity of which is not consistent with the applicable product information (e.g., Investigator’s Brochure for an unapproved investigational medicinal product).

**12.5 Serious adverse event (SAE)**

An SAE is any untoward medical occurrence that at any dose of study medication may have resulted in:

- Death
- An immediate risk of death (i.e., “life threatening”)
- Admission to a hospital as an inpatient or prolongation of an existing hospitalization
- A persistent or significant disability or incapacity
- A congenital anomaly or birth defect
- A medical event that, based on clinical judgment, may jeopardize the patient and may require intervention to prevent one of the outcomes listed above

All Grade 4 severity events listed on the Gilead-modified NIAID Common Toxicity Tables for Adults (Appendix D) will be considered SAEs. Grade 3 severity events will be evaluated on a case-by-case basis and may be reported as SAEs if warranted in the clinical judgment of the evaluating physician. However, signs and symptoms will only be reported themselves as SAEs when a diagnosis of the event cannot be established and itself reported as an SAE.

**12.6 Evaluation of adverse events**

For every possible AE identified by interview, examination, or laboratory result, the study clinicians will evaluate and document on the appropriate study forms the following information:

- Nature: A description of the event or condition
- Severity: Graded in terms of interference with usual social and functional activities
- Mild: No or minimal interference with usual social and functional activities
- Moderate: Greater than minimal interference with usual social and functional activities
- Severe: inability to perform usual social and functional activities
- Life-threatening: inability to perform basic self-care functions; or medical or operative intervention indicated to prevent permanent impairment, persistent disability, or death
- Death
- Expectedness: In light of information in the Investigator’s Brochure
- Relationship: The relation between an adverse event and study drug may be characterized by grades of likeliness:
- Not related: clearly explained by another, documented cause
- Probably not related: more likely explained by another cause than by study drug
- Possibly related: study drug and other cause explain event equally well
- Probably related: more likely explained by study drug than any other cause
- Definitely related: Direct association with study drug
- Seriousness: Determination of whether it meets the definition of an SAE (listed above)
- Requirements for clinical management: Determine whether to suspend use of study medication, temporarily or permanently; conduct any indicated diagnostic or confirmatory testing; administer or refer for any indicated treatment; determination of need for unblinding to deliver effective care

**12.7 Reporting of safety events**

**12.7.1 Expedited reporting**

Prompt reporting by email to CDC will be done for all AEs which are unexpected, serious, and may be related to study drug use (i.e., possibly, probably, or definitely related by above listed definitions) within 2 working days of CDC staff awareness of the incident. It will be initiated within 72 hours of the site’s awareness of the event by faxing a preliminary report and completed within 10 days by faxing a complete report to all of the following:

Deputy Permanent Secretary Thailand MOPH – U.S. CDC Collaboration

Chairperson BMA ERC DDC7 Building, 4^th^ Floor

Bangkok Metropolitan Administration Nonthaburi 11000, Thailand

173 Dinso Road, Bangkok 10200 Tel: 0-2580-0669

Tel: 0-2221-4866, 0-2226-4871 Fax: 0-2580-0712

Fax: 0-2221-4866

Dr. Terry Chorba Global Drug Safety Associate

Human Subjects Contact Gilead Sciences

National Center for HIV, STD, and TB Research Fax: +1-650-522-5477

U.S. Centers for Disease Control and Prevention Phone: +1-650-522-5894

Fax: +1-404-639-8600 E-mail: [Safety_FC@gilead.com](mailto:Safety_FC@gilead.com)

Phone: +1-404-639-8000

E-mail: [anv3@cdc.gov](mailto:anv3@cdc.gov)

Dr. Chris Elias The Ethical Review Committee for Research

Program for appropriate Technology in Health in Human Subjects

Chairperson, DSMB Ministry of Public Health

Fax: +1-206-285-6619 Nonthaburi 11000, Thailand

Phone: +1-206-285-3500 Fax: +66-2591-8251

E-mail: [celias@path.org](mailto:celias@path.org) Phone: +66 2 591 8251, 66 2 590 6172

Director

Drug Control Division

Food and Drug Administration

Ministry of Public Health

Tivanon Road

Nonthaburi 11000

Fax: +66-2590-7165

Phone: +66-2590-7060

**12.7.2 Routine periodic reporting**

Only routine reporting will be done for all ADRs, defined as AEs which may be related to study drug use (i.e., possibly, probably, or definitively related by above listed definitions) and that do not meet the criteria for SAE.

Routine reporting will consist of periodically providing a line listing of the events by type (e.g., lab toxicity or clinical event). These listings will be provided to the Gilead Sciences, Inc., U.S. CDC, and Institutional IRBs/ERCs for each annual continuation review; for each DSMB review; and on request from any regulatory agency involved in these trials.

**12.7.3 Documented on clinical forms but not otherwise reported**

All AEs will be documented on the appropriate clinical study forms. Those which are judged “unrelated” to study medication or “unclassifiable” (using the above listed criteria) will not be reported routinely, but the data will be available in the database should reporting of specific conditions be requested in future. Examples of such conditions are: accidental injuries (e.g., resulting from motor vehicle accidents or fires), diagnosed infectious diseases (e.g., malaria, urinary tract infection, STIs), progression of pre-existing conditions (e.g., cervical dysplasia).

**12.8 Clinical management of adverse events**

Adverse clinical events will be evaluated and managed at presentation by study physicians. The study physician will provide initial treatment and/or referral to an appropriate clinic or hospital. A decision will be made about the continuation of study product based on the toxicity grading scale (Appendix D) using the instructions outlined in section 12.8.1.

**12.8.1 Management of laboratory abnormality *(except serum creatinine elevation)* or clinical event**

**Grade 1 or 2:** Continue study medication at the discretion of the study physician.

**Grade 3 or 4:** May continue study medication, at the discretion of the study physician, for up to 3 days while values are confirmed.

**Confirmed grade 3:** If unrelated to study drug by above definitions, may continue dosing. If causal relationship to study drug possible by above definitions, withhold drug until toxicity returns to < grade 3 then retest to confirm participant is HIV negative then restart study drug. If toxicity > grade 3 recurs, permanently discontinue dosing and follow at scheduled study visits until toxicity resolves.

**Confirmed grade 4:** If unrelated to study drug by above definitions, may continue dosing. If causal relationship to study drug possible by above definitions, permanently discontinue study drug and follow at scheduled study visits until toxicity resolves. Do not rechallenge.

**12.8.2 Management of serum creatinine elevation**

The baseline serum creatinine is defined as the last value obtained from the laboratory prior to the administration of the first dose of study drug.

**All grades:** Continue study medication for up to 3 days while serum creatinine value is confirmed.

**Confirmed grade 1:** Continue study medication at the discretion of the study physician and monitor as clinically indicated, generally weekly until serum creatinine value is within normal limits (Grade 0).

**Confirmed grade 2-4:** Permanently discontinue study drug and monitor as clinically indicated, generally weekly until serum creatinine value returns to within normal limits (Grade 0).

**12.8.3 Management of creatinine clearance results**

Study medication will be adjusted according to modified Tenofovir dose adjustment for renal impairment guidelines (Gilead Sciences).^10^

|  | Creatinine Clearance | | |
| --- | --- | --- | --- |
|  | ≥50 mL/min | 30–49 mL/min | < 29 mL/min |
| Recommended Dosing Interval | Every 24 hours | Every 48 hours | Stop study medication |

**13. Statistical summary**

The aim of this study is to determine if daily oral tenofovir 300 mg prevents HIV infection and is safe among IDUs in Thailand.

**13.1 Safety outcome**

Based on the AIDSVAX B/E vaccine trial, conducted 1999-2003 among IDUs enrolled from the same 17 BMA drug treatment clinics, we would expect a baseline SAE rate of about 7% during the year participants take study drug (Unpublished data). Assuming a sample size of 1000 per arm, table 3 shows the power to detect incremental increases in reported SAEs among tenofovir recipients compared to the placebo recipients using a 2-tail test and a 0.05 level of significance.

Table 3. Power to detect differences in reported SAEs among placebo and tenofovir recipients.

| Placebo Recipients | Tenofovir Recipients | Power |
| --- | --- | --- |
| 7% | 8% | 11% |
| 7% | 9% | 34% |
| 7% | 10% | 64% |
| 7% | 11% | 86% |
| 7% | 12% | 96% |
| 7% | 13% | 99% |

In summary, we have 80% power to detect an SAE rate above 11% among tenofovir recipients (4% greater than the 7% expected among placebo recipients).

**13.2 Effectiveness outcome**

Efficacy analysis is designed to determine if tenofovir efficacy to prevent HIV infection exceeds a threshold level of 10%. A lower threshold of 10% was selected in order to provide a result that can guide public health actions. HIV incidence during the AIDSVAX B/E HIV vaccine trial was 3.4 per 100 person-years. We suspect HIV incidence among IDUs in Bangkok has declined due to HIV prevention activities of BMA drug treatment clinic staff and changes in drug use (i.e., less heroin, more midazolam and methamphetamines)^5^. In order to ensure that there is sufficient power to achieve the study’s primary objective, to determine if tenofovir prevents HIV infection, we will use an endpoint-driven study design. Based on simulations assuming tenofovir efficacy of 50%, 67%, and 90% and a 1-tail alpha of 0.025, we determined power to provide a result with a lower limit 95% CI > 10% (Table 4).

Table 4. Power to demonstrate tenofovir efficacy with 95% CI >10%

| Estimated Tenofovir Efficacy | Expected Endpoints by Group | | | Power to show tenofovir efficacy with lower limit 95% CI > 10% |
| --- | --- | --- | --- | --- |
|  | Tenofovir | Placebo | Total |  |
| 50% | 12 | 24 | 36 | 37% |
| 67% | 9 | 27 |  | 78% |
| 90% | 3 | 33 |  | 99% |
| 50% | 13 | 27 | 40 | 49% |
| 67% | 10 | 30 |  | 82% |
| 90% | 4 | 36 |  | 99% |
| 50% | 14 | 30 | 44 | 54% |
| 67% | 11 | 33 |  | 86% |
| 90% | 4 | 40 |  | >99% |

If tenofovir efficacy is 67%, we have 82% power to demonstrate at least 10% tenofovir efficacy with 40 endpoints. A human post-exposure prophylaxis study suggests tenofovir efficacy to prevent HIV infection will exceed 67%.^8^

**13.3 Measures to minimize bias**

Multiple methods will be used to reduce bias:

- Randomization of participants to treatment arms
- Blinding of treatment arms (participants, study staff, data analysts)
- Using ACASI to ask risk behavior and adherence questions
- Immediate tracking for missed visits

**13.4 Randomization**

Study participants will be randomly assigned (1:1) to receive tenofovir or placebo using permuted blocks. The study arm assignment list will be generated by an independent statistician and the assignment codes will be known only to the statistician, Gilead, and the DSMB.

**13.5 Blinding**

Gilead Sciences, Inc. will supply matching placebo and tenofovir tablets. Tablet bottles will also be labeled with the participant ID so study clinic staff can dispense medication corresponding to the assigned treatment arm while remaining blinded. Using this method the participants, study clinicians, data management staff, monitors, statisticians, and investigators actively involved in the trial will not know which participants are taking tenofovir and which are taking placebo.

**13.6 Unblinding procedure**

The randomization code will be held by a statistician at CDC, Atlanta and a person to be identified at Gilead Sciences, Inc. Unblinded information will be provided to the principal investigator if needed in an emergency to guide treatment decisions for any participant. At the conclusion of the study, when primary analyses are complete and the study is unblinded to the investigators, participants will be offered the opportunity to learn into which arm they were randomized.

**13.7 Analysis plan summary**

All randomized patients will be included in the analysis. The analysis will be performed on an intention-to-treat basis. Data from participants lost to follow-up will be censored at the last contact. To assess the success of randomization, a summary of baseline variables by study arm will be tabulated. Continuous data will be summarized with descriptive statistics, including n, mean, standard deviation, minimum, maximum, median and interquartile range. Categorical data will be summarized using counts and percents. A significance level alpha=0.05 will be used to perform significance testing and construct confidence intervals. Heterogeneously distributed variables will be regarded as potential confounders and will be adjusted for in multivariable modeling. Missing data will be summarized by treatment groups, and missing patterns will be assessed using graphical and modeling approaches and an appropriate multiple imputation technique will be applied.^24^ All analyses will be done using SAS version V8 (Cary, NC, U.S).

**13.8 Analysis of safety outcomes**

AEs, SAEs, and abnormal laboratory test results will be compared between the study arms. Factors that may be associated with AEs will be analyzed. AEs will be organized by frequency, severity, and relationship to study agent in each study arm. Rates of AEs among tenofovir and placebo recipients will be compared using chi-squared tests. Longitudinal analysis of AEs will be examined using generalized estimating equation (GEE) logistic regression. The time to adverse event will be examined using Kaplan-Meier plots, log-rank tests, and Cox proportional hazards models to compare the incidence of AEs.

**13.9 Efficacy analysis**

The study hypothesis is that HIV incidence will be lower among participants receiving tenofovir than among participants receiving placebo. A log-rank statistic will be used to test the efficacy of treatment.^25^ The efficacy of treatment will be estimated as

Efficacy = 1 – *RR*

where *RR* is the hazard ratio estimated from a discrete time proportional hazards regression model with an indicator variable representing treatment arm. Confidence intervals for efficacy can be calculated using the confidence limits for the *RR*.

All analyses will be performed on an intention-to-treat basis. Rates of HIV seroconversion with Poisson 95% exact confidence intervals will be used to summarize the data. Discrete time proportional hazards regression will be applied to adjust the treatment effect for age and gender and for other demographic or behavioral variables, which, despite the randomization, were heterogeneously distributed between the study arms.

**13.10 Interim analysis plan summary**

A safety analysis will be conducted when approximately 200 person-years of participant observation are accrued. Assuming SAE rates among placebo recipients are similar to rates in the AIDSVAX B/E trial (i.e., 7/100 person-years), we calculated power to detect differences in relative rates of SAEs with 200 person-years of follow-up time (100 in each arm) using a 2-tail test at the 0.05 level of significance using methods described elsewhere.^26^

Table 5. Power to detect differences in relative rates of SAE reports at 200 person-year follow-up.

| Expected Placebo Rate | Expected Tenofovir Rate | Relative Rate | Power |
| --- | --- | --- | --- |
| 7/100 | 14/100 | 2.0 | 32% |
| 7/100 | 17.5/100 | 2.5 | 46% |
| 7/100 | 21/100 | 3.0 | 71% |
| 7/100 | 24.5/100 | 3.5 | 83% |
| 7/100 | 28/100 | 4.0 | 91% |
| 7/100 | 31.5/100 | 4.5 | 95% |
| 7/100 | 35/100 | 5.0 | 97% |

We have 83% power to detect an SAE rate of 24.5 per 100 person-years or higher (RR > 3.5) at the first safety evaluation.

Additional safety analyses will be conducted annually. Efficacy analyses will be conducted when approximately 24 seroconversions have accrued and at the end of the study (40 seroconversions). A group sequential design will be applied to handle multiplicity in analyses. The DSMB will be advised to continue/prematurely stop the trial based on O’Brien-Fleming stopping boundary^27^ using an alpha-spending function approach where the overall type-1 error rate of 0.025 (1-tail) can be spent in a flexible manner^28^. As the trial has been designed as an end-point driven trial for a total of 40 seroconversion, we will use direct monitoring to control type-1 error (0.025); using EAST software (EAST v5, Cytel Software, Cambridge MA, 2007) and the alpha spending function as specified. The cut-off points for the 1-tail log-rank Z-test at the interim and final analyses will be:

- First interim analysis (60% of total events): Z > 2.669 or p < 0.003804 (1-tail)
- Final analysis: Z > 1.981 or p < 0.023796 (1-tail)

If the point estimate of efficacy at the interim analysis is <20%, a conditional power futility analysis^29^ will be carried out. If the conditional power futility analysis shows less than 10% power to demonstrate a lower 95% confidence interval of tenofovir efficacy of >10% given the observed data and using the original trial assumptions of event rates for future observations in the remaining person-years to be accrued, the DSMB will consider recommending trial termination.

The interim analysis report will include a list of AEs, missed visits, drop-outs, adherence information, protocol violations and number, percentage and rates of occurrence of primary and secondary outcomes. Frequencies and crude rates with 95% confidence intervals will be used for data summarization. If necessary, adjustment of sample size will be performed. Multivariable analysis will adjust for baseline differences between study arms.

**14. Monitoring plan**

**14.1 Clinical monitoring plan**

Site assessment and site monitoring will be performed during the trial to:

- Verify compliance with human subjects and other research regulations and guidelines
- Assess adherence to study protocol, study-specific procedures manual, and local counseling practices
- Confirm the quality and accuracy of information collected at the study sites and entered into the study database

The Principal Investigator will allow study monitors to inspect study facilities and documentation (e.g., informed consent forms, clinic and laboratory records, other source documents, case report forms), as well as observe the performance of study procedures. The Principal Investigator will also allow inspection of all study-related documentation by authorized representatives of the CDC, Gilead Sciences, Inc., the study monitor, and U.S. and in-country government and regulatory authorities. A site visit log will be maintained at the study site to document all visits.

**14.2 Safety monitoring plan**

The Principal Investigator is responsible for continuous close monitoring of all AEs that occur among study participants, and for alerting study staff if unexpected concerns arise. The DSMB will have members from Botswana, Thailand, and the United States (sites of CDC supported tenofovir HIV prophylaxis trials). Members will be selected based on demonstrated expertise in statistics, medical ethics, epidemiology, behavioral science, medical care of HIV-infected individuals, and clinical research experience. The DSMB will be responsible for periodic (or emergency) review of safety data, and decisions about holding accrual or administration of study product for safety concerns.

Prior to initiation of the trial a data safety monitoring plan will be written and agreement about the plan reached with the DSMB. It will include at least the following:

- Specification of the adverse event detection and reporting system
- Content of interim data reports
- Frequency of interim data reviews
- Statistical methods for interim analyses
- Specification of safety and efficacy stopping rules
- Distribution of interim review reports

Interim reports will include at least the following information, by treatment arm, gender and age:

- Number randomized
- Number and timing of missed visits and drop-outs, accumulated follow-up time
- Baseline characteristics of study population
- Medication adherence
- Protocol violations, including ineligible persons enrolled, number who received an unassigned treatment, instances of unblinding
- Safety and efficacy outcomes
- number, percent, and rate of occurrence of primary outcomes
- number, percent, and rate of occurrence of secondary outcomes
- line listing of adverse events

Interim reports will also include:

- Multivariate analyses of outcomes adjusted for baseline differences between arms/strata
- Analyses of efficacy outcomes adjusted for medication adherence

At predetermined review intervals, the board will be convened (in person or by phone) to review the data provided and make a recommendation about:

- - Whether sufficient safety is demonstrated in the phase II trial to proceed to phase III
  - Whether safety and efficacy interim data indicate the continuation, revision, or discontinuation of the phase III trial

**14.3 Protocol compliance monitoring plan**

Every attempt will be made to conduct the study in full compliance with the approved protocol. Ongoing monitoring of staff compliance with protocol procedures will be performed on-site by the study monitor, reviewed by the Principal Investigator, and corrective actions taken (and documented).

**14.4 Emergency protocol violations**

When a departure from protocol is crucial to protect the safety and well-being of an individual participant, it may be instituted for that patient only and the Principal Investigator will be informed of the event immediately. The Principal Investigator will promptly notify all IRBs/ERCs in writing including documentation of the reasons for deviation from the protocol, the outcome for the patient, and if any changes in the protocol are indicated based on this event.

**14.5 Non-emergency protocol violations**

Significant departures from the protocol that result from staff error that are not urgent safety concerns for participants will also be promptly reported to the Principal Investigator and a memo written to document the occurrence and any corrective actions which have been taken or are recommended to prevent a recurrence. Copies of these memos will also be sent to all IRBs/ERCs. Examples of these violations include:

- Omission or improper administration of informed consent
- Enrolling ineligible participants
- Treatment errors, either failure to deliver indicated treatment or administration of incorrect treatment
- Incorrectly timed or omitted study procedures and assessments
- Coercing a participant to remain in the study after they express a desire to withdraw
- Failure to discontinue study medication or discontinue a participant when indicated by the protocol
- Evidence of falsified data by any member of the study staff

**15. Data management plan**

**15.1 Data management and analysis software**

Data management will be done using a Microsoft SQL 2000 database system (Microsoft Corp, Redmond, WA) with an audit log as required by FDA (21 CFR part 11) for management of clinical trial data. All data collection forms (e.g., CRFs) and laboratory data will be double-entered at TUC into this database (e.g., enrollment, HIV test results). QDS, version 2.1 or later (Nova Research, Bethesda, MD) will be used to program the ACASI screens for collection of risk behavior and adherence data. An exportable database will be produced containing participant responses to questionnaire items which will be imported into the main database software. QDS data files will be sent to TUC as a password protected zip file via email or file transfer via the internet on a regular basis.

Microsoft Access and other development tools such as Visual Basic (VB), and VB Script (Microsoft Corp, Redmond, WA) will be used to develop double data entry systems which will interface effectively with the SQL database, allowing export and import of other relevant data in a part 21 CFR part 11 compliant manner. Once data has been double-entered or imported into the master database, all subsequent data changes will be tracked by an electronic audit log that records details of each change (e.g., user ID, date of change, reason for change). Other data requirements will include documenting staff training/annual retraining and managing inventory of study product, disposables, and test kits.

Data analysis will be done using SAS for Windows, version 8 (SAS Institute, NC). DBMSCOPY v8 (DataFlux, Cary, NC) will be used to convert the data from MS SQL format to SAS format.

**15.2 Quality assurance of data collection forms**

Procedural, face-to-face interview, clinical examination, and laboratory test result data will be entered onto paper forms by study staff. Clinic physicians will review laboratory result forms and determine whether clinical follow-up is indicated. All other forms, and laboratory result forms after review, will be sent to data entry staff for data entry. Quality control reports and queries will be routinely generated and distributed to clinic staff to verify data and resolve queries.

**15.3 Data storage**

The Principal Investigator will assure that complete, accurate, and current study records data are maintained and stored in a secure manner throughout the study. In accordance with U.S. federal regulations, the Principal Investigator will retain all study records for at least 5 years after the conclusion of the study.

**16. Administrative procedures**

**16.1 Study initiation**

The following documents will be in place at the Principal Investigator’s office before any potential participants are contacted:

- Signed agreement between Gilead Sciences, Inc. and CDC
- CVs for trial investigators, study coordinators, laboratory coordinators
- Signed protocol and attachments
- Sample CRFs
- Information given to participants (e.g., consents, educational materials, recruitment materials)
- Instructions for handling of investigational product and other trial related medications
- Decoding procedures for blinded trials
- IRB/ERC approvals and composition
- Thai FDA approvals
- Laboratory accreditations
- Normal laboratory test values
- Financial disclosure forms for each trial investigator
- Financial records
- Shipping records
- Site visit log

**16.2 Study conduct**

During the conduct of the study, any revisions to the above listed documents and additional documents listed here will be in place and available for review by monitors:

- Documentation of investigational product and trial-related materials shipment
- Investigational products accountability documents
- Relevant communications (other than site visits)
- Signed consent forms
- Source documents
- Completed CRFs with documentation of CRF corrections
- Participant screening log/listing
- Participant enrollment log/listing
- Staff signature list
- SAE notifications
- Interim or annual reports to IRBs, ERCs, regulatory authorities, and CDC
- Progress reports to U.S. CDC

**17. Study coordination**

The BTSG in collaboration with TUC will serve as the coordination and operations center for study development and implementation. Gilead Sciences, Inc. will provide tenofovir and placebo for the study. Assignment of all sponsor responsibilities for this study will be specified in a Clinical Trials Agreement executed by CDC and Gilead Sciences, Inc.

**17.1 Study completion**

After completion of the trial, all documents in sections 16.1 and 16.2 will be maintained in files together with the following:

- Documentation of investigational product destruction (if destroyed at the sites)
- Complete participant ID code list
- Final report by investigator to IRBs, ERCs, regulatory authorities, Gilead Sciences, Inc., and CDC.

**17.2 Site record retention and access to documents at the site**

Study records will be stored on-site until data entry is completed, database queries resolved, and records have been externally audited by study monitors. They will then be archived and stored in secure facilities for at least 5 years after study completion.

**18. Laboratory specimens and biohazard containment**

**18.1 Laboratory testing**

Clinic staff will be trained and their proficiency tested for the performance and interpretation of oral fluid HIV tests (OraQuick Rapid HIV Antibody test) and urine pregnancy tests. Small on-site laboratories at each study clinic will be used to complete oral fluid HIV tests and urine pregnancy tests. Clinic staff will be responsible for the labeling, packaging, and arranging transport of specimens to laboratories. Specimens will be shipped in accordance with International Air Transport Association, U.S. CDC, and BMA specimen shipping regulations.

The BMA laboratory, located in downtown Bangkok, will be responsible for completing chemistry, haematology, HIV-1 EIA, HIV-1 Western Blot, and hepatitis serology testing. The TUC laboratory, on the MOPH campus, will be responsible for completing HIV-1 subtype determination, CD4 counts, HIV viral load levels, HIV antiretroviral resistance testing, PBMC – plasma separation, and other tests as appropriate.

Each study-associated laboratory will adhere to standards of good laboratory practice and local standard operating procedures (SOPs) for proper collection, processing, labeling, transporting, and storing specimens.

**18.2 Specimen storage and possible future research testing**

Study participants will be asked to provide written informed consent for their specimens to be stored after the end of the study for possible future testing. Specimens will be stored at the TUC laboratory for 20 years to allow long term safety testing, if necessary. Any future proposals to use specimens for tests that are not related to HIV or AIDS will be submitted for review to the MOPH and BMA Ethical Review Committees and the CDC Institutional Review Board. There are no plans to use these specimens for commercial purposes. The specimens of participants who do not consent to long-term storage will be destroyed at the end of the study.

**18.3 Biohazard containment**

As the transmission of HIV and other blood-borne pathogens can occur through contact with contaminated needles, blood, and blood products, appropriate blood and secretion universal precautions will be employed by all personnel in the drawing of blood, and shipping and handling of all specimens for this study.

**19. References**

1. Weniger B, Limpakarnjanarat K, Ungchusak K et al. The epidemiology of HIV infection in Thailand. AIDS 1991;5(suppl 2):S71-S85.

2. [Phoolcharoen W, Ungchusak K, Sittitrai W, Brown T.](http://www.ncbi.nlm.nih.gov:80/entrez/query.fcgi?cmd=Retrieve&db=pubmed&dopt=Abstract&list_uids=9679638) Thailand: lessons from a strong national response to HIV/AIDS. AIDS 1998;12:S123-35.

3. Vanichseni S, Kitayaporn D, Mastro TD, et al. Continued high HIV-1 incidence in a vaccine trial preparatory cohort of injection drug users in Bangkok, Thailand. AIDS 2001;15:397-405.

4. Vanichseni S, Tappero JW, Pitisuttithum P, Subhachaturas W, Kitayaporn D, Raktham S, van Griensven F, Mastro TD, Tantikul T, Vimutisunthorn E, Suntharasamai P, Kaewkungwal J, Migasena S, Gee CC, Heyward WL, Francis DP, Choopanya K, for the Bangkok Vaccine Evaluation Group. Recruitment, screening, and epidemiologic characteristics of participants in the AIDSVAX B/E HIV vaccine efficacy trial, Bangkok, Thailand. AIDS 2004; 18:311-316.

5. Van Griensven F, Kaewkungwal J, Vanichseni S, Tappero JW, Sangkom U, Hiranrus K, Kitayaporn D, Pitisuttithum P, Mastro TD, Suntharasamai P, Natrujirote P, Phasithiphol B, Luck A, Orelind K, Heyward WL, and Choopanya K, for the Bangkok Vaccine Evaluation Group. Lack of increased HIV risk behavior among injection drug users participating in the AIDSVAX B/E HIV vaccine efficacy trial in Bangkok, Thailand. AIDS 2004; 18:295-301.

6. [Mastro TD, Kitayaporn D, Weniger BG, Vanichseni S, Laosunthorn V, Uneklabh T, Uneklabh C, Choopanya K, Limpakarnjanarat K.](http://www.ncbi.nlm.nih.gov:80/entrez/query.fcgi?cmd=Retrieve&db=pubmed&dopt=Abstract&list_uids=8017531) Estimating the number of HIV-infected injection drug users in Bangkok: a capture–recapture method. Am J Public Health 1994;84:1094-9.

7. Klausner RD, Fauci AS, Corey L, Nabel GJ, Gayle H, Berkley S, Haynes BF, Baltimore D, Collins C, Douglas RG, Esparza J, Francis DP, Ganguly NK, Gerberding JL, Johnston MI, Kazatchkine MD, McMichael AJ, Makgoba MW, Pantaleo G, Piot P, Shao Y, Tramont E, Varmus H, Wasserheit JN. The need for a global HIV vaccine enterprise. Science 2003;300:2036-9.

8. Cardo DM, Culver DH, Ciesielski CA, et al. A case-control study of HIV seroconversion in health care workers after percutaneous exposure. N Engl J Med 1997;337:1485-90.

9. Youle M and Wainberg MA. Pre-exposure chemoprophylaxis (PREP) as an HIV prevention strategy. J Int Assoc Physicians AIDS Care. 2003;2:102-105.

10. Gilead Sciences. Investigator’s Brochure: Viread^TM^ Tenofovir Disoproxil Fumarate (PMPA Prodrug) (GS-4331-05) Eighth Edition; October 31, 2002.

11. Smith PF, Kearney BP, Liaw S, et. al. Effect of Tenofovir Disoproxil Fumarate on the Pharmacokinetics and Pharmacodynamics of Total, R-, and S-Methadone. Pharmacotherapy 2004;24:970-7.

12. Martin M, Mansakul W, Tappero J, et al. Care and Treatment of HIV-infected Injecting Drug Users During an HIV Vaccine Efficacy Trial, Bangkok, Thailand. XV International AIDS Conference, Bangkok Thailand 2004. Poster MoPeB3350.

13. Celentano DD, Vlahov D, Cohn S, Shadle VM, Obasanjo O, Moore RD. Self-reported antiretroviral therapy in injection drug users. JAMA 1998;280:544-6.

14. Mellors JW, Kingsley LA, Rinaldo CR, et al. Quantitation of HIV-1 RNA in plasma predicts outcome after seroconversion. Ann Int Med 1995;122:573-9.

15. Palmer S, Margot N, Gilbert H, et al. Tenofovir, adefovir, and zidovudine susceptibilities of primary human immunodeficiency virus type I isolates with non-B subtypes or nucleoside resistance. AIDS Res Hum Retroviruses. 2001;17:1167-1173.

16. Mondy K and Tebas P. Emerging bone problems in patients infected with human immunodeficiency virus. CID 2003;36 (supple2):S101-105.

17. Castillo AB, Tarantal AF, Watnik MR and Martin RB. Tenofovir treatment at 30 mg/kg/day can inhibit cortical bone mineralization in growing rhesus monkeys. J Orthop Res 2002;20:1185-1189.

18. Barditch-Crovo P, Deeks SG, Collier A*, et al.* Phase I/II trial of the pharmacokinetics, safety, and antiretroviral activity of tenofovir disoproxil fumarate in human immunodeficiency virus-infected adults. *Antimicrob Agents Chemother* 2001,45:2733-2739.

19. Strathdee SA, Palepu A, Cornelisse PGA, Yip B, O’Shaughnessy MV, Montaner JSG, Schechter MT, Hogg RS. Barriers to use of free antiretroviral therapy in injection drug users. JAMA 1998;280:547-9.

20. Sherer R. Adherence and antiretroviral therapy in injection drug users. JAMA 1998;280:567-8.

21. Peyiere H, Reynes J, Rouanet I, et al. Renal tubular dysfunction associated with tenofovir therapy: report of 7 cases. J Acquir Immune Defic Syndr 2004;35:269-73.

22. Rollot F, Nazal EM, Chauvelot-Moachon L, et al. Tenofovir-related Fanconi syndrome with nephrogenic diabetes insipidus in a patient with acquired immunodeficiency syndrome: the role of lopinavir-ritonavir-didanosine. Clin Infect Dis 2003;37:e174-6.

23. Karras A, Lafaurie M, Furco A, et al. Tenofovir-related nephrotoxicity in human immunodeficiency virus-infected patients: three cases of renal failure, Fanconi syndrome, and nephrogenic diabetes insipidus. Clin Infect Dis 2003;36:1070-3.

24. Carpenter J, Pocock S, Lamm CJ. Statistics in Medicine 2002; 21:1043-1066.

25. Prentice RL, Gloeckler LA. Regression analysis of grouped survival data with applications to breast cancer data. Biometrics 1978;34:57-67.

26. Clayton D, Hills M. Statistical Models in Epidemiology. Oxford, Oxford Universtity Press; 1993. p. 205-211.

27. O’Brien PC, Fleming TR. A multiple testing procedure for clinical trials. Biometrics 1979;35:549-56.

28. Lan KKG, DeMets DL. Discrete sequential boundaries for clinical trials. Biometirka 1983;70:659-663.

29. Jennison C and Turnbull BW. Group Sequential Methods: Applications to Clinical Trials. Chapman-Hall 2000.

**Appendix A.**

Site number ___ ___ ___ Subject number___ ___ ___ ___

**Study of the Safety and Efficacy of Daily Tenofovir to Prevent HIV Infection Among Injection Drug Users in Bangkok, Thailand (Bangkok Tenofovir Study)**

**Screening Consent Form**

(Flesch-Kincaid Reading Grade Level 7.3)

**Introduction**

The Bangkok Metropolitan Administration and the Centers for Disease Control and Prevention in the United States are doing a research study in Bangkok. The first goal of this study is to learn if a medicine (tenofovir) works to prevent infection by the virus that causes AIDS (HIV). The second goal is to see if it is safe when used by injection drug users in Bangkok. This medicine is usually used along with other medicines to fight HIV in people who are already infected. We do not know if tenofovir will prevent HIV infection and it is not currently approved for this use. We are doing this study to see if it does.

To see if you are eligible to join the study, we will ask you to:

- Answer a few questions
- Have some counseling about HIV tests
- Allow us to take blood and urine samples and swab your mouth for testing
- Have some counseling about how to lower your chances of getting or passing HIV
- Return to the clinic in 1-2 weeks

We call this process screening. To help you decide if you will be screened today, please understand that:

- It is your choice to be screened or not
- If you wish to be screened, but not today, you can come back at another time
- If you decide not to be screened, you will still receive all regular care you get now at the BMA clinics
- You will be given information about HIV testing and asked to sign a form giving your permission for an HIV test to be done for you today
- If you give permission for the test, you will get the results of your HIV test today.

**What do I need to do if I agree to be screened?**

First, we will ask you some questions about yourself, including some questions about your drug use history.

If you are a woman, we will collect a small amount of urine to check if you are pregnant.

Then we will tell you about HIV tests and answer any questions you have about them. We will ask permission to do an HIV test today from a swab of your mouth. If you give permission, we will do the test while you wait.

If your test shows you may have the HIV virus now, we will take blood to do a second test to check whether you have the HIV virus. But you will not be eligible for the study we will be doing. We will make an appointment for you to come back to the clinic in 1-2 weeks to talk with a counselor about the test results.

If your test shows that you do not have the HIV virus now, we will take blood (about 2 tablespoons) to check your health. We will tell you more about the study and answer your questions. We will ask you to come back in 1-2 weeks when we have results from all the blood tests. It will take about 2 hours to complete screening.

**Will being screened benefit me?**

If you agree to be screened, you will learn your HIV status and other things about your health. You will get education and counseling about ways to keep from getting or passing HIV. You will be given free condoms and contraceptives and taught how to use them. If you have HIV infection or another health problem, we will help you get care at a BMA hospital or clinic.

**are there any risks to being screened?**

The questions we will ask you about drug use may make you feel uneasy. Blood drawing may cause some discomfort, bleeding or bruising, lightheadedness, and rarely, infection. If your blood test shows that you have HIV infection now, it may be upsetting to you. We will help you if you get upset by giving counseling when you get your test result and at later visits if you want.

**How will my information be protected?**

All study-related information will be treated as confidential. To monitor the study and the safety of volunteers, Trial Ethical Committees, study monitors, and regulators will have access to volunteer medical records. We will use a code number, not your name, on interview forms, blood and urine samples we take for testing, and test results. The code number we give you will be used to keep track of all your records. Consent forms and information you give us to contact you will be kept locked up away from all the other study forms. Only the study leaders will have a list with both names and code numbers.

**Is there any cost for being screened?**

There is no cost to you for screening, blood tests, or counseling. If you agree to be screened today, you will receive 350 baht (8.75 USD) for travel costs, time, and effort you will spend being screened.

**What are my rights if I agree to be screened?**

You will be informed if information becomes available that might effect your decision to join the study. If you have any questions or concerns about whether you should join this study, you can talk to Khun Suchada Akarangsi (tel. 02 247 9736). Khun Suchada works at the Drug Abuse Prevention and Treatment Division of the BMA. Khun Suchada is not involved in the study, but she knows about it. She will be glad to help answer your questions.

It may be that you have questions about your rights as a study subject or that you think you have been harmed because you were in this study. In this case you can contact Dr. Premwadee Karahadej, Secretary of the BMA Ethical Review Committee (tel: 081 822 9178).

You may refuse to answer questions or give blood or urine at any time during the study. If you have questions or concerns about the study, please call and speak to Dr. Udomsak Sangkum (tel: 089 762 3777).

If you agree to be screened, you will be agreeing to do several things, as best you can including:

- Answer the questions asked
- Give permission for blood and urine and a swab of your mouth to be taken
- Give permission for an HIV test
- Receive the results of your HIV test today
- If you are eligible, consider being in the HIV prevention study

**Statement of Consent**

I have read or have had this form read to me. I have been told the purpose of the screening, what will be done, and the risks and benefits of being screened. By signing below, I agree to be screened.

______________________ __________________ _______________

Volunteer Name Signature Date

______________________ __________________ _______________

BMA Representative Name Signature Date

The study volunteer cannot read. I verify that this consent form has been accurately and clearly read to the study volunteer.

______________________ _________________ _______________

Witness Name Signature Date

**Storage of samples**

We are asking your consent to store your leftover blood samples. These samples may be used for future testing, including molecular biological studies, to test for diseases and conditions which may be associated with HIV and AIDS. Because we will have removed all identifying information, we will not be able to give you the results.

Samples will be stored at the TUC laboratory for 20 years to allow long term safety testing, if necessary. Any future proposals to use your samples for tests that are not stated in this protocol will be submitted for review to the MOPH and BMA Ethical Review Committees and the CDC Institutional Review Board. If you do not want us to store your samples, you can still be in this study. If you agree to have your samples stored but change your mind later, you can tell the clinic staff at any time. We will then destroy your samples.

Please **sign** the following:

[ ] I agree to have my leftover blood specimens stored for future testing.

[ ] I do not agree to have my leftover blood specimens stored for future testing.

__________________ ______________________ _______________

Volunteer Name Signature Date

______________________ __________________ _______________

BMA Representative Name Signature Date

The study volunteer cannot read. I verify that this consent form has been accurately and clearly read to the study volunteer.

______________________ _________________ _______________

Witness Name Signature Date

**Appendix B.**

Site number ___ ___ ___ Subject number___ ___ ___ ___

**Study of the Safety and Efficacy of Daily Tenofovir to Prevent HIV Infection Among Injection Drug Users in Bangkok, Thailand (Bangkok Tenofovir Study)**

**Enrollment Consent Form**

(Flesch-Kincaid Reading Grade Level 7.9)

**Introduction**

The Bangkok Metropolitan Administration and the Centers for Disease Control and Prevention in the United States are doing a research study in Bangkok. The first goal of this study is to learn if a medicine (tenofovir) works to prevent infection by the virus that causes AIDS (HIV). The second goal is to see if it is safe when used by injection drug users in Bangkok.

Before deciding whether to be in the study, it is important that you understand that:

- Being in the study is voluntary
- If you join the study but later change your mind, you may stop at any time
- If you decide not to be in the study, or if you stop, you will still receive all regular care you get at the BMA clinics.

**Why are we doing this study?**

People who inject drugs with needles used by others or have sex can catch HIV, the virus that causes AIDS. Using condoms and not injecting with needles used by others are way we have to prevent this. But some people do not use condoms every time they have sex or use clean needles every time they inject. So, we are trying to find new ways to help prevent HIV infection. One of the new ways is for people who don’t have HIV infection to take a medicine that fights the virus. This medicine, called tenofovir, is usually used along with other medicines to fight HIV in people who are already infected. We do not know if tenofovir will prevent HIV infection and it is not currently approved for this use. We are doing this study to see if it does.

Because drug users often get HIV infection, we are looking for drug users to join the study so we can test tenofovir and find out if it prevents HIV infection. There will be 2,400 volunteers in this study.

We can only test medicines to prevent HIV infection in people who do not have it. Since you have tested negative for the virus, and are healthy, we would like you to join this study.

**What you need to do if you join the study?**

If you agree to be in the study, we will ask you to come for visits until everyone finishes the study. Most people will come for visits for 12 to 24 months. If it takes more than 36 months to enroll 2,400 volunteers, people who enrolled first may be asked to come for 36 to 48 months. You can choose to come to clinic everyday to take your medicine or once a month to see a counselor.

***First visit (Enrollment)***

At your first visit we will teach you how to answer some questions using a computer. These questions are different than the ones you had for screening. We will also do a physical exam, an HIV test and, for women, a urine pregnancy test.

We will assign you to one of two groups. You have an equal chance to be in either group. One group will get tenofovir, the medicine that may prevent HIV infection. The other group will get a medicine that looks exactly the same but doesn’t have any effect and is called a placebo. Until the study is over, we won’t know, and the people in the study won’t know, who is taking which medicine. We do it this way so that we won’t let our hopes about the medicine influence the way we collect the information. We will talk to you about how to take the medicine.

We will give you counseling about how to lower your chances of getting HIV infection. We will give you free condoms and contraceptives. This is important for two reasons. We don’t know who is getting tenofovir. Also we don’t know yet if it works well to protect people from getting HIV.

We will give you appointments for your next set of study visits. This first visit will last about 2 hours.

***Follow-up Visits***

Every month when you come back we will ask you some questions about:

- How well you are doing at taking the pills every day
- How well you are doing at lowering your chances of getting HIV
- How you are feeling physically

We will also

- Collect the bottle and leftover pills we gave you at the last visit and give you a new bottle
- Talk with you about how to handle any problems you are having with taking the pills
- Take a swab of your mouth to do an HIV test
- Do a urine pregnancy test for women.

At the first two monthly visits we will take 2 tablespoons of blood. After that we will take 2 tablespoons of blood every 3 months. Blood will be tested for HIV and to see if you are anemic or have other medical problems. The monthly visits will take about 1 hour.

Every three months we will do all the monthly visit things and also ask you to use the computer to answer questions. We will counsel you about how to lower your risk of HIV infection. And we will take 2 tablespoons of blood. These visits every three months will take about 2 hours.

If you become HIV infected during the study, we will ask you to come for visits at 1 month, 2 months, and every 4 months, months after infection until the study ends. These visits will take about 2 hours. We will talk to you about HIV and answer your questions. We will ask you to have a pregnancy test (women). We will take about 2 tablespoons of blood at each visit. We will ask you to use a computer to answer questions each 4 months.

If you are placed in jail/prison during the study, we will come to the jail/prison to see you for follow-up visits. Being in the study will not affect your condition in jail/prison or the likelihood of parole.

It is important to take the pills every day. In addition to the regular follow-up visits, you can choose to come to clinic every day for a very short visit. At this visit, you will take your pill and we will make a record of this. This visit will take 2 to 3 minutes.

| **Study Visit** | **What will happen** |
| --- | --- |
| **Enrollment visit** | Answer questions on computer  Answer questions with a person  Have an oral HIV test  *Women*: urine pregnancy test  Get assigned to a group  Be given pills and told how to take them  Get counseling to lower HIV risk |
| **Follow-up visits**  Every month      Every three months | Answer questions with a person  Bring in unused pills, bottle, and med diary  Get counseling about taking pills  Get new pills  Have an oral HIV test  Give a blood sample (first 2 months only)  *Women****:*** urine pregnancy test  Same as monthly visit, plus:  Answer questions on computer  Give a blood sample |

**Are there special study procedures for women?**

- Women agree to take a urine pregnancy test at monthly visits.
- We do not know if tenofovir is safe for a fetus. Because of this, during the study women must not have sexual intercourse or, if they have sexual intercourse, must use birth control (oral, injection, or barrier). Information on birth control methods will be given and contraceptives will be provided free of charge.
- If a woman becomes pregnant during the trial, she agrees to stop taking the study drug. She will be referred for ante-natal care. Women agree to allow clinic staff to see them every 3 months during their pregnancy to check on her health and the health of her child/children at birth.

**Will being in the study benefit me?**

If you join the study, you will learn your HIV status and other things about your health. You will get education and counseling about ways to keep from getting HIV. You will be given free condoms, contraceptives and bleach and taught how to use them. If you have HIV infection or another health problem while you are in the study, we will help you get care at a BMA hospital or clinic. If we find out tenofovir is safe and prevents HIV infection we will give tenofovir at no cost to all volunteers for at least 1 year. During this year, staff will be available at the clinic every day to give you tenofovir. At monthly visits, we will do an oral HIV test and a urine pregnancy test (for females). At months 3, 6, 9, and 12, we will do the HIV and pregnancy tests and ask you to answer questions on a computer and give blood for testing. You will not receive compensation for the daily or monthly visits but will receive 350 baht for visits at months 3, 6, 9, and 12.

**Are there any risks to being in the study?**

The questions we will ask you about sexual and drug use behaviors and time you might have spent in jail or prison may make you feel uneasy. However, you do not have to answer any question that makes you feel uncomfortable. You can stop answering the questions at any time. Blood drawing may cause some discomfort, bleeding or bruising, make you feel light headed and rarely infection. If any test shows that you have gotten the HIV virus while you are in the study, it may be upsetting to you. We will help you if you get upset by giving counseling before and after your test result and at later visits if you want.

If you get tenofovir, there is a small chance that it will upset your stomach, give you diarrhea, make you vomit, or hurt your kidneys. It is also possible that your bones will store less calcium. But in studies with people who took tenofovir with other medicines to fight HIV, when this happened it didn’t affect their health. We will check closely with lab test for kidney or bone problems.

If you get HIV infection while you are taking tenofovir, there is a very small chance that the virus might learn to fight off the medicine. We will do an HIV test at every clinic visit so we can stop the study medication quickly if you get infected to try and prevent this.

If others find out that you are part of a study about HIV in drug users, they may think you are at risk for or have HIV infection. They may also react negatively to you in other ways. It is possible that others may learn your HIV status. We will do everything we can to prevent this.

**How will my information be protected?**

All study-related information will be treated as confidential. To monitor the study and the safety of volunteers, Trial Ethical Committees, study monitors, and regulators will have access to volunteer medical records. We will use a code number, not your name, on interview forms, blood and urine samples we take for testing, and test results. The code number we give you will be used to keep track of all your records. All records will be stored in locked files at the BMA clinic. Your name will never be used in any publication or presentation about the study. Consent forms and information you give us to contact you will be kept locked up away from all the other study forms. Only the study leaders will have a list with both names and code numbers.

We will ask you how to reach you if you miss a study visit. We will only contact you in the way you tell us.

**Is there any cost for being in the study?**

There is no cost to you for examinations, blood tests, or counseling. If you agree be in the study, you will receive 350 baht (8.75 USD) for every monthly visit. This is to make up for the cost of travel, time, and effort. If you choose daily visits, we will give you 70 baht (1.90 USD) for each visit and another 350 baht each week that you come all 7 days.

**Reasons you may be taken out of the study without your consent**

You may be taken out of this study if you fail to come to your scheduled appointments or if you become too sick to continue. The sponsor or other authority could also end the study.

**What are the alternatives to participation?**

If you do not wish to join this study, HIV testing and counseling are available at the BMA clinic or other clinics in your area. However, you may have to pay for these services by yourself.

**What are my rights if i agree to be in the study**

You will be informed if information becomes available that might effect your decision to join or continue the study. If you have any questions or concerns about whether you should join this study, you can talk to Khun Suchada Akarangsi (tel. 02 247 9736). Khun Suchada works at the Drug Abuse Prevention and Treatment Division of the BMA. Khun Suchada is not involved in the study, but she knows about it. She will be glad to help answer your questions.

It may be that you have questions about your rights as a study subject or that you think you have been harmed because you were in this study. In this case you can contact Dr. Premwadee Karahadej, Secretary of the BMA Ethical Review Committee (tel: 01 822 9178).

You may refuse to answer questions or give blood or urine at any time during the study. If you have questions or concerns about the study, please call and speak to Dr. Udomsak Sangkum (tel: 089 762 3777).

In case of emergency, you can call any of the following researchers: Dr. Kachit Choopanya, the Principle Investigator (tel. 081 644 2554), Dr. Pirapong Saicheua, of the BMA Medical Services Department (tel. 089 833 0484), Dr. Montira Thongsari, of the BMA Department of Health (tel. 081 939 8661), or Dr. Udomsak Sangkum (tel: 089 762 3777).

We will also give you the name and phone number of the study coordinator at the local BMA clinic in case you have questions about the study.

If you agree to be in the study, you will be agreeing to do several things, as best you can including:

- Come to the clinic for visits every month for at least 12 months
- Come to the clinic daily if you join the every day visit schedule
- Answer the questions asked at each visit
- Take the pills you are given every day
- Have blood drawn at the first three monthly visits, then every 3 months
- Have an oral HIV test at each monthly visit
- Give us a way to contact you if you miss a study visit

**Statement of Consent**

I have read or have had this form read to me. I have been told the purpose of the study, what will be done, and the risks and benefits of enrollment. I have been told being in the study is completely voluntary. I can stop at any time and this will not effect services I get at BMA clinics. By signing below, I agree to be enrolled.

______________________ __________________ _______________

Volunteer Name Signature Date

______________________ __________________ _______________

BMA Representative Name Signature Date

The study volunteer cannot read. I verify that this consent form has been accurately and clearly read to the study volunteer.

______________________ _________________ _______________

Witness Name Signature Date

**Storage of samples**

We are asking your consent to store your leftover blood samples. These samples may be used for future testing, including molecular biological studies, to test for diseases and conditions which may be associated with HIV and AIDS. Because we will have removed all identifying information, we will not be able to give you the results.

Samples will be stored at the TUC laboratory for 20 years to allow long term safety testing, if necessary. Any future proposals to use your samples for tests that are not stated in this protocol will be submitted for review to the MOPH and BMA Ethical Review Committees and the CDC Institutional Review Board. If you do not want us to store your samples, you can still be in this study. If you agree to have your samples stored but change your mind later, you can tell the clinic staff at any time. We will then destroy your samples.

Please **sign** the following:

[ ] I agree to have my leftover blood specimens stored for future testing.

[ ] I do not agree to have my leftover blood specimens stored for future testing.

__________________ ______________________ _______________

Volunteer Name Signature Date

______________________ __________________ _______________

BMA Representative Name Signature Date

The study volunteer cannot read. I verify that this consent form has been accurately and clearly read to the study volunteer.

______________________ _________________ _______________

Witness Name Signature Date

**Appendix C. Bangkok Tenofovir Study Laboratory Specimen Testing**

| **Test** | **Specimen** | **Visits** | **Assay** | **Assay site** |
| --- | --- | --- | --- | --- |
| HIV infection | Oral transudate (swab) | Screening, enrollment, and monthly visits | OraQuick (Orasure) | On-site clinic lab |
| HIV confirmatory testing | SST tube (9.5 ml) | OraQuick reactive only | WB, EIA (Biorad) | BMA Lab |
| Chemistries* |  | Screening visit, M1, M2 and every 3 months | Automated analyzer | BMA Lab |
| HBV serology** |  | Screening visit | EIA (Dade, Berhing) |  |
| CBC | EDTA tube (5 ml) | Screening visit, M1, M2 and every 3 months | Automate Analyzer | BMA Lab |
| CD4 | EDTA tube (5 ml) | All seroconverters | Flow cytometry (BD) | TUC lab |
| HIV plasma viral load |  |  | Amplicor Monitor (Roche) |  |
| HIV-1 subtyping |  |  | Peptide serotyping (CDC) |  |
| Pregnancy test | Urine (women) | Screening, enrollment and monthly visits | Rapid β-HCG (Commercial Science) | On-site clinic labs |
| HIV antiretroviral resistance testing | EDTA tube (5mL) | All seroconverters | TruGene HIV-1 genotyping  (Visible Genetics) | TUC Lab |
| TDF levels | serum | All seroconverters in TDF arm and random 10% of non-seroconverters | HPLC | To be determined |
| HIV sequencing | PBMC | All seroconverters | Sequencing | TUC Lab |

*Chemistries: BUN, creatinine, electrolytes, ALT, AST, bilirubin, amylase, phosphorus, calcium, alkaline phosphatase

**hepatitis B virus surface antigen (HbsAg), anti-HBs IgG, anti-HB core IgG

**Appendix D. Gilead Modified Common Toxicity Grading Scale – Adults**

IV = intravenous

|  | **Grade 1** | **Grade 2** | **Grade 3** | **Grade 4** |
| --- | --- | --- | --- | --- |
| **SERUM CHEMISTRY** | | | | |
| Creatinine (mg/dL) | An increase   0.5 from baseline | 2.1 – 3.0 | 3.1 – 6.0 | >6.0 |
| BUN (mg/dL) | 30 – 60 | 61 – 120 | 121 –240 | >240 |
| Magnesium (mEq/L) | 1.0 - <1.2 | 0.9 - <1.0 | - | - |
| Phosphorus (mg/dL) | 2.0 – 2.2 | 1.5 – 1.9 | 1.0 – 1.4 | <1.0 |
| Glucose (mg/dL) | 55 – 64 or  116 – 160 | 40 – 54 or  161 – 250 | 30 – 39 or  251 – 500 | <30 or  >500 |
| Bilirubin (mg/dL) | 1.3 – 1.8 | 1.9 – 3.0 | 3.1 – 6.0 | >6.0 |
| Amylase (U/L) | 97 – 131 | 132 – 175 | 176 – 439 | >440 |
| Lipase (U/L) | 154 – 210 | 211 – 279 | 280 – 699 | >699 |
| Creatine Kinase (U/L)  *(unrelated to trauma, exercise, or IM injection)*  Male  Female | 248 – 498  211 – 423 | 499 – 990  424 – 845 | 991 – 1980  846 – 1690 | >1980  >1690 |
| Cholesterol (mg/dL) | >ULN – 300 | 301-400 | 401-500 | >500 |
| Triglycerides (mg/dL) | - | 400 – 750 | 751 – 1200 | >1200 |
| Sodium (mEq/L) | 130 - <131 or  148 – 150 | 123 -129 or  151 – 157 | 116 -122 or  158 – 165 | <116 or  >165 |
| Potassium (mEq/L) | 3.0 – 3.3 or  5.6 – 6.0 | 2.5 – 2.9 or  6.1 – 6.5 | 2.0 – 2.4 or  6.6 – 7.0 | <2.0 or  >7.0 |
| Bicarbonate (mEq/L) | 16 - <17 | 11-15.9 | 8 – 10.9 | <8 |
| Calcium (mg/dL)  *(Ionized)*  Calcium (mg/dL)  *(corrected for albumin)* | 3.0 – 3.4 or  5.6 – 6.0  7.8 - <8.4 or  10.6 – 11.5 | 2.5 – 2.9 or  6.1 – 6.5  7.0 – 7.7 or  11.6 – 12.5 | 2.0 – 2.4 or  6.6 – 7.0  6.1 – 6.9 or  12.6 – 13.5 | <2 or  >7.0  <6.1 or  >13.5 |

|  | **Grade 1** | **Grade 2** | **Grade 3** | **Grade 4** |
| --- | --- | --- | --- | --- |
| **For patients with normal LFTs at baseline (< 2x ULN)** | | | | |
| AST or SGOT (U/L)  *Male*  Female | 45 – 90  43 – 85 | 91 – 180  86 – 170 | 181 – 360  171 – 340 | >360  >340 |
| ALT or SGPT (U/L)  *Male*  *Female* | 54 – 108  43 – 85 | 109 -215  86 – 170 | 216 – 430  171 – 340 | >430  >340 |
| Alkaline Phosphatase (U/L)  *Male 15-20 y*  *Male 21-59 y*  *Female 15-59 y* | 313 – 625  138 – 275  138 – 275 | 626 – 1250  276 – 550  276 – 550 | 1251 – 2500  551 – 1100  551 – 1100 | >2500  >1100  >1100 |
| **HEMATOLOGY** | | | | |
| Hemoglobin (g/dL) | 8.0 – 9.4 | 7.0 – 7.9 | 6.0 – 6.9 | <6.0 |
| Absolute Neutrophil Count (cells/mm^3^) | 1000 – 1500 | 750 – 999 | 500 – 749 | <500 |
| Platelets  (cells/mm^3^) | 75,000 – 100,000 | 50,000 – 74,999 | 25,000 – 49,999 | <25,000 or  diffuse petechiae |
| PT (seconds) | 15 – 19 | 20 – 22 | 23 – 45 | >45 |
| APTT (seconds) | 37 – 61 | 62 – 86 | 87 – 111 | >111 |

|  | **Grade 1** | **Grade 2** | **Grade 3** | **Grade 4** |
| --- | --- | --- | --- | --- |
| **URINE** | | | | |
| Hematuria | Microscopic only  or  1 – 10 RBCs | Gross hematuria, no clots  or  10 – 100 RBCs | Gross hematuria with clots or  >100 RBCs  or too numerous to count | Obstructive or treatment required |
| Proteinuria  (dipstick)  (mg/dL) | 1+  30 – 99 | 2+ to 3+  100 – 1000 | 4+  >1000 | Nephrotic syndrome |
| Glycosuria (dipstick) | 1+ | 2+ | 3+ | 4+ |
| **CLINICAL FINDINGS** | | | | |
| **Cardiovascular** | | | | |
| Dyspnea | Dyspnea on significant exertion | Dyspnea at normal level of activity | Dyspnea at rest | Requires ventilatory support |
| Cardiac Function | - | Symptoms with ordinary activity  Slight limitation of activity | Symptoms with less than ordinary physical activity  Marked limitation of activity | Symptoms with any activity or even at rest |
| Hypertension | 20 – 25 % increase from baseline systolic | 26 – 30 % increase from baseline systolic | 31 – 40 % increase from baseline systolic | >40 % increase from baseline systolic |
| Hypotension | 20 – 25 % decrease from baseline systolic | 26 – 30 % decrease from baseline systolic | 31 – 40 % decrease from baseline systolic | >40 % decrease from baseline systolic |
| **Gastrointestinal** | | | | |
| Oral Discomfort/Stomatitis | Mild discomfort,  no difficulty swallowing | Difficulty swallowing but able to eat and drink fluids | Unable to swallow solids | Unable to drink fluids, requires IV fluids |
| Diarrhea | Mild or transient, 3–4 loose stools in 24 hours ,OR mild diarrhea lasting < 1 week | Moderate or persistent, 5-7 loose stools in 24 hours, OR 3-4 loose stools per day >1 week | Severe, bloody diarrhea or 8-9 loose stools in 24 hours, or orthostatic hypotension | Life threatening, hypotensive shock, OR hospitalization for IV fluids |

|  | **Grade 1** | **Grade 2** | **Grade 3** | **Grade 4** |
| --- | --- | --- | --- | --- |
| Nausea | Mild or transient, maintains reasonable food intake | Moderate discomfort, some limitation of food intake | Severe discomfort, minimal food intake for 3 or more days | Life threatening, unable to ingest any food or fluid in 72 hours |
| Vomiting | Mild or transient, 2-3 episodes in 24 hours OR 1-2 episodes per day lasting <1 week | Moderate or persistent, 4-5 episodes in 24 hours OR 1-2 episodes per day for > 1 week | Severe, vomiting of all food/fluids in 24 hours OR orthostatic hypotension | Life threatening, hypotensive shock |
| Abdominal Pain | Mild, occasional, transient | Moderate, transient, no or minimal treatment required | Severe or requiring analgesia | Severe with guarding or peritoneal signs |
| **Neuro/Neuromuscular** | | | | |
| Headache | Mild, no therapy required | Moderate, non-narcotic analgesic therapy required | Severe, responds to initial narcotic therapy | Intractable, requiring repeated narcotic therapy |
| Peripheral Neuropathy | Mild discomfort,  no therapy required | Moderate discomfort persisting for >72 hours, non-narcotic analgesia required OR mild discomfort persisting for >72 hours accompanied by loss of deep tendon reflex previously present | Severe discomfort, marked antalgic gait, narcotic analgesia required with symptomatic improvement | Incapacitating, intolerable discomfort, not improved OR unable to walk despite narcotic analgesia |
| Mood/Neuropsych | Mild anxiety or  mild depression | Therapy required for moderate anxiety or moderate depression | Needs assistance for  severe anxiety or  severe depression or  severe mania | Acute psychosis or incapacitated or hospitalization |
| Muscle Strength | Subjective weakness;  no objective symptoms | Mild objective weakness; no decrease in function | Objective weakness;  function limited | Paralysis |

|  | **Grade 1** | **Grade 2** | **Grade 3** | **Grade 4** |
| --- | --- | --- | --- | --- |
| Myositis | Minimal findings | Patient must have some measures of myositis (positive EMG or muscle biopsy) and one of the following:  mild to moderate muscle pain for >4 weeks;  may require nonsteroidal anti-inflammatory drugs  OR  Difficulty climbing stairs or rising from a sitting position but able to ambulate without assistance | Patient must have some measures of myositis (positive EMG or muscle biopsy) and one of the following:  Moderate to severe muscle pain for >4 weeks requiring nonsteroidal anti-inflammatory drugs  OR  Needs some assistance with ambulation or general activities | Patient must have some measures of myositis (positive EMG or muscle biopsy) and one of the following:  Muscle weakness; inability to ambulate, requiring special care and assistance with mobilization  OR  Acute rhabdomyolosis, muscle necrosis and edema, moderate to severe muscle weakness with inability to ambulate or mobilize without assistance  OR  Acute rhabdomyolosis with electrolyte imbalance in renal failure |
| Neuro Cerebellar | Slight incoordination; dysdiadochokinesia | Intention tremor; dysmetrial; nystagmus | Locomotor ataxia | Incapacitated |

|  | **Grade 1** | **Grade 2** | **Grade 3** | **Grade 4** |
| --- | --- | --- | --- | --- |
| **Skin/Allergy** | | | | |
| Allergic Reaction | Prurititis without rash | Localized urticaria | Generalized urticaria, angioedema | Anaphylaxis |
| Local Reaction | Erythema OR tenderness | Induration <10 mm or phlebitis or inflammation | Induration 10 mm or ulceration | Necrosis of skin |
| Cutaneous | Erythema OR pruritis | Diffuse maculopapular rash, dry desquamation | Vesiculation, moist desquamation, ulceration | Exfoliative dermatitis, mucous membrane involvement, OR  Stevens-Johnson, OR erythema multiforme, OR necrosis requiring surgery |
| **Other Measurements** | | | | |
| Fatigue | Normal activity reduced <25% | Normal activity reduced 25–50% | Normal activity reduced >50%, cannot work | Unable to care for self |
| Fever  (Centigrade)  (Fahrenheit) | 37.7 – 38.5°  100.0 – 101.5° | 38.6 – 39.5°  101.6 – 102.9° | 39.6 – 40.5°  103.0 – 105.0° | >40.5°  > 105.0° |
| Any Other Toxicity Not  In the Table | Transient or mild discomfort; requiring no limitation of activity; and no therapy required | Mild to moderate impact on activity; requiring some assistance and medical intervention | Marked impact on activity; requiring some assistance and medical intervention | Complete disability; requiring significant assistance and medical intervention and/or hospitalization |

**Appendix E. HIV testing algorithm**

Collect Oral fluid specimen

OraQuick

Give same-day result in clinic

non-reactive

Collect blood and make appointment for participant to return for result in 1 week

reactive

Send blood to BMA lab for EIA and WB (if necessary)^1^

1. AIDSVAX B/E vaccine trial participants, if they choose, may be screened for enrollment in the Bangkok Tenofovir Study. Approximately 50% of vaccine trial participants received the AIDSVAX B/E vaccine, a gp-120 subunit vaccine. In order to ensure that we provide participants with correct information, we will do WB testing on EIA positive specimens to determine if EIA positive results are due to HIV or vaccine.
